# When Do Trait‐Based Higher Order Interactions and Individual Variation Promote Robust Species Coexistence?

**DOI:** 10.1002/ece3.71336

**Published:** 2025-04-25

**Authors:** Gaurav Baruah, György Barabás, Robert John

**Affiliations:** ^1^ Faculty of Biology, Theoretical Biology University of Bielefeld Bielefeld Germany; ^2^ Division of Biology Linköping University Linköping Sweden; ^3^ Institute of Evolution, Centre for Ecological Research Budapest Hungary; ^4^ Department of Biological Sciences, Center for Climate and Environmental Studies IISER Kolkata India

**Keywords:** Eco‐evolutionary dynamics, higher order interactions, intraspecific variation, species coexistence, stability, trait clustering

## Abstract

Models on the effects of individual variation often focus on pairwise interactions, but communities could harbor both pairwise and higher order interactions (HOIs). Theoretical studies on HOIs, where a third species modulates pairwise species competition, tend to assign them at random, even though they could be mediated and structured by one‐dimensional traits. Here, we consider two different classes of models of both pairwise and higher order trait‐mediated interactions: competition alleviated by increasing trait distance, and hierarchical competition where species higher in the hierarchy exert more competition on those lower and vice versa. Combining these models with evolutionary dynamics based on quantitative genetics, we compare their impact on species diversity, community pattern, and robustness of coexistence. Regardless of individual variation, trait‐mediated HOIs generally do not promote and often hinder species coexistence, but there are some notable exceptions to this. We present an analytical argument to make sense of these results and argue that while the effects of trait‐based HOIs on diversity may appear confusing on the surface, we can understand what outcome to expect in any given scenario by looking at the shape of the effective interaction kernel that arises from the joint action of pairwise and HOI terms. In addition, we find that (i) communities structured by competitive trait hierarchies are highly vulnerable to external perturbations, regardless of HOIs, and (ii) trait‐based HOIs with distance‐dependent competition create the most robust communities, with minimal impact from individual variation, and (iii) both individual variation and HOIs consistently lead to a more even distribution of species traits than would occur by chance. These findings suggest that trait‐mediated HOIs foster coexistence only under special conditions, raising the question of whether HOIs must involve multiple traits to positively affect coexistence in competitive communities.

## Graphical abstract

Higher order interactions mediated by phenotypic traits could foster coexistence only under very special conditions, raising the question of whether HOIs must involve multiple traits to positively affect coexistence in competitive communities.

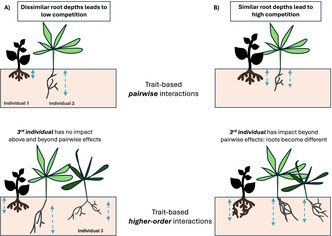

## 1 Introduction

Explanations for multispecies coexistence in ecological communities have largely been sought at the species level by emphasizing mean differences among species driven by competitive interactions or life‐history trade‐offs (Gravel et al. 2011; Violle and Jiang 2009; Clark et al. 2010; Kraft et al. 2015; Valladares et al. 2015). The idea is that differences among species along multiple ecological dimensions would minimize niche overlap and permit long‐term species coexistence (MacArthur and Levins 1967). However, species often compete for only a small number of limiting resources (Hutchinson 1961; Laird and Schamp 2006; Li and Chesson 2016; Shoresh et al. 2008; Letten and Stouffer 2019), and numerous species may coexist despite little differences in demographic or resource‐based niches (Condit et al. 2006), which poses a challenge for classical coexistence models. Such classical competition models of coexistence consider interactions between every possible pairing of species and require precise parameter trade‐offs to stabilize communities or to limit the strength of competition among coexisting species in accordance with the competitive exclusion principle (MacArthur and Levins 1967; Vandermeer 1975). Theoretical studies with competition models further show that any stability achieved through strong self‐regulation or strong intraspecific pairwise competitive interactions (Barabás et al. 2017) can be disrupted by strong interspecific interactions among species.

Competitive interactions among species are not always constrained to species pairs and can involve higher order combinations (Wilson 1992; Laird and Schamp 2006; Grilli et al. 2017; Mayfield and Stouffer 2017), where interactions between a species pair are modulated by one or more species. In an ecological system where pairwise interactions structure communities, higher order interactions (HOIs) may modify these pairwise interactions and restructure communities (Levine et al. 2017; Singh and Baruah 2021). For example, a superior competitor for a limiting resource will inhibit an inferior competitor for the same resource, but a third species may modulate the strength of this inhibition without affecting either of the two competitors directly (Bairey et al. 2016). Such attenuation of the pairwise inhibitory effect can be density‐mediated or trait‐mediated and can lead to qualitatively different community dynamics compared to the case with purely pairwise interactions (Grilli et al. 2017). HOIs that have been studied thus far are mostly parameterized by assigning interaction coefficients that are drawn from normal distributions of the interaction values (Bairey et al. 2016; Letten and Stouffer 2019). With such random HOI structures, species coexistence is promoted provided the coefficients fulfill certain conditions (Singh and Baruah 2021). Random HOIs could be termed “high‐dimensional,” because when a diversity of different independent species traits determine interaction coefficients, their values can often be modeled as being effectively random and independent of one another. By contrast, low‐dimensional HOIs could come into effect when interactions are structured by just a handful of species traits. Indeed, in communities, species interactions are dictated by the local neighborhood and the traits species possess (Guimarães et al. 2011; Maruyama et al. 2014; McPeek 2017; Baruah et al. 2018), which consequently could structure communities and impact stability (Barabas and D'Andrea 2016; Baruah 2022). While the importance of such HOIs has been recognized (Levine et al. 2017), the nuances of such structured HOIs and competitive pairwise interactions in communities have proven difficult to study theoretically or empirically.

The emphasis on pairwise species interactions in studies of coexistence is potentially limiting not just because pairwise interactions disregard HOIs but also because of its focus on species and treating them as homogeneous units. Indeed, within‐species trait variation is often ignored from species coexistence mechanisms (Siefert 2012; Hart et al. 2016). It is now clear that intraspecific variation can have both ecological and evolutionary effects on competitive interactions, which ultimately determine patterns of species coexistence (Yamamichi et al. 2022; Pastore et al. 2021). For example, intraspecific trait variation can undermine species coexistence by increasing or decreasing competitive ability, niche overlap, and even spacing among species (Barabas and D'Andrea 2016), or by altering competitive outcomes through nonlinear averaging of performances (Hart et al. 2016). There is equally compelling evidence that intraspecific variation promotes species coexistence whenever species have similar mean traits but different intraspecific trait variances (Bolnick et al. 2011; Barabas and D'Andrea 2016). Experimental work has shown that although intraspecific variation allows a community to be resilient against invaders, it creates an opportunity for competitive exclusion among strong competitors (Hausch et al. 2018). Empirical studies in the field have found, for instance, that leaf economic traits such as specific leaf area, leaf N, and P consistently display high intraspecific variation (Meziane and Shipley 1999) and influence community assembly and ecosystem functioning (Reich 2014). Given that high levels of intraspecific trait variation within communities appear to be more a rule than an exception, the implications of intraspecific variation to community structure merit detailed investigation.

Clearly, both higher order species interactions and intraspecific variation can have a significant influence on community structure, but these two properties have always been investigated separately in studies on species coexistence. The effects of intraspecific trait variation and eco‐evolutionary dynamics on structuring large communities where both pairwise and HOIs dominate a community are therefore unexplored. Theoretical analyses indicate that purely pairwise interactions in a community lead to more even trait spacing than expected by chance (D'Andrea and Ostling 2016; Barabas and D'Andrea 2016). However, a community dominated by both pairwise and HOIs could lead to greater trait clustering along a trait axis. The apparent mechanism could be that with intraspecific variation present, HOIs can alleviate and stabilize the negative pairwise interactions that would otherwise lead to distinct spacing. One of our main goals here is to see whether and when this is in fact the case.

In this study, we examine the importance of trait‐mediated structured HOIs and intraspecific variation in promoting species coexistence and shaping community structure. We do this by modeling a one‐dimensional quantitative trait that contributes to the pairwise and higher order competitive abilities of phenotypes interacting in the community. Next, we formulate four models of how structured trait‐mediated HOIs could influence pairwise competition. In modeling phenotype‐mediated species competition, we consider both distance‐alleviated (Gaussian) competition where being more different in traits leads to diminished competition, as well as hierarchical competition in which species with better traits exert a competitive dominance on those with inferior traits, but not vice versa (Adler and Mosquera 2000). An example of the latter could be tree height in competition for light, where taller individuals shade shorter ones without receiving much competition in turn (Pacala et al. 1996). We find that structured trait‐based HOIs most often do not increase, and can even decrease, species diversity, but that there are some exceptions to this—especially whenever the interaction kernel corresponding to pairwise effects has a large width, but the one corresponding to HOIs is narrow. Given these results, we also present a heuristic analytical argument to clarify when and why the addition of trait‐based HOIs promotes coexistence. It turns out that this can be understood in terms of the combined effective pairwise interaction kernel that results after averaging higher order effects over all species (Gibbs et al. 2022). An upshot of this analysis is that trait‐based HOIs seem to promote diversity under possibly atypical circumstances only. Our results open up a discussion on whether higher order interactions could then facilitate species coexistence in high(er) dimensional trait spaces (Grilli et al. 2017; Fox 2023; Singh and Baruah 2021; Kleinhesselink et al. 2019).

## 2 Methods and Models

### 2.1 Modeling Framework

Our modeling framework focuses on understanding species coexistence within a competitive community dictated by higher order interactions, where competition is determined by species traits along a one‐dimensional trait axis. Previous studies have modeled species coexistence similarly in competitive communities but with competition primarily driven by pairwise species interactions (Barabas and D'Andrea 2016; Pastore et al. 2021; Kremer and Klausmeier 2013). In such models, individuals of a species vary along a unidimensional trait of interest. The distribution of this trait is considered under the quantitative genetic limit, assuming it is controlled by a very large number of loci, each with a small additive effect. As a result, the trait distribution remains normal, with their variance constant despite selection acting on the mean trait value (Bulmer 1980; Barton et al. 2017). In our competitive community model, we consider S competing species composed of phenotypes z along a one‐dimensional trait axis. Each locus contributes a small additive amount to the phenotype z. The distributions of phenotypes $p_{i}\left(z\right)$ of the species are then normal with mean $u_{i}$ and variance $\sigma_{i}^{2}$. Competition happens in two ways. First, there are pairwise effects: two phenotypes with trait values z and $z^{\prime }$ compete with one another depending on the difference in their traits. Second, there may also be higher order effects where the presence of a third phenotype ${z^{\prime }}^{\prime }$ may modify the interaction between two phenotypes z and $z^{\prime }$. The per capita growth rate of phenotype z is given by

(1) $$\begin{array}{cc} r\left(z\right) & =r_{0}\left(z\right)-\sum_{j=1}^{S}N_{j}\int a\left(z,z^{\prime }\right)p_{j}\left(z^{\prime }\right)\;dz^{\prime }\\ & -\sum_{j=1}^{S}\sum_{k=1}^{S}\;N_{j}N_{k}\iint W\left(z,z^{\prime },{z^{\prime }}^{\prime }\right)p_{j}\left(z^{\prime }\right)p_{k}\left({z^{\prime }}^{\prime }\right)\;dz^{\prime }d{z^{\prime }}^{\prime }, \end{array}$$

where $r_{0}\left(z\right)$ is the intrinsic growth rate of phenotype z, $N_{j}$ is the population density of species j, $a\left(z,z^{\prime }\right)$ is a pairwise interaction kernel measuring the competitive effect of phenotype $z^{\prime }$ on phenotype z, the integration extends from minus to plus infinity, and $W\left(z,z^{\prime },{z^{\prime }}^{\prime }\right)$ is a higher order interaction kernel mediating three‐way interactions at the phenotype level. As higher order interactions are determined by the same unidimensional trait as the pairwise ones, they are structured and low‐dimensional in nature.

To truly represent higher order interaction effects, any higher order kernel should in principle satisfy the property $W\left(z,z,z\right)=0$. Indeed, a “higher order effect” refers to how the presence of a phenotype disrupts or enhances the interaction between two others. But if the three phenotypes are exactly the same, then $W\left(z,z,z\right)$ simply amounts to an intraspecific (or, in our case, intra‐phenotype) reduction in per capita growth rates, which ought to be part of the phenotype's self‐limitation and not of higher order interactions. In practice however, it suffices for $W\left(z,z,z\right)$ to be a constant. This is because then every phenotype experiences the exact same degree of self‐limitation, so it becomes a question of taste whether one considers this as part of the HOIs or as a separate, common density‐dependent term (Section S1.2 in Data S1). But a higher order kernel that explicitly depends on z even when all three phenotypes are equal introduces hidden self‐regulatory effects, which can spuriously enhance coexistence. For this reason, we make sure below that our models satisfy the criterion that $W\left(z,z,z\right)$ is a z‐independent constant.

The growth rates of Equation (1) are turned into an eco‐evolutionary system by substituting them into the pair of equations

(2) $$\frac{dN_{i}}{dt}=N_{i}\int r\left(z\right)p_{i}\left(z\right)\;dz$$

and

(3) $$\frac{du_{i}}{dt}=h_{i}^{2}\int \left(z-u_{i}\right)r\left(z\right)p_{i}\left(z\right)\;dz$$

(Barabas and D'Andrea 2016; Pastore et al. 2021), where $h_{i}^{2}$ is the heritability of the trait for species i. Equation (3) refers to the evolutionary dynamics of the one‐dimensional mean trait, $u_{i}$, in response to selective pressure on the mean trait of a species i due to the position in the trait axis, and due to selective pressure caused by competition from other species (in either a pairwise or a higher order setting). The integral in the equation is analogous to the selection gradient $\partial r\left(z\right)/\partial u_{i}$, and in fact reduces to it in the limit of small intraspecific variation $\sigma_{i}$. This then yields the often‐used evolutionary formula $du_{i}/dt=h_{i}^{2}\sigma_{i}^{2}\left(\partial r\left(z\right)/\partial u_{i}\right)$ (e.g., Schreiber et al. 2011; McPeek 2017). Equation (3) is a generalization that applies even when $\sigma_{i}$ is not small.

By specifying the functions $r_{0}\left(z\right)$, $a\left(z,z^{\prime }\right)$, and $W\left(z,z^{\prime },{z^{\prime }}^{\prime }\right)$ in Equation (1) and substituting the resulting per capita growth rates into Equations (2) and (3), we get a full eco‐evolutionary model. We now classify four different models that emerge from making different choices for these functions:

#### 2.1.1 Model 1: Resource‐Mediated Competition Model (evo)

In this model, HOIs are absent and so $W\left(z,z^{\prime },{z^{\prime }}^{\prime }\right)=0$. Pairwise interactions are derived from an explicit model of resource consumption in which the interaction strength between two phenotypes z and $z^{\prime }$ turns out to be a decreasing (Gaussian) function of their difference:

(4) $$a\left(z,z^{\prime }\right)=\exp\left(-\frac{{\left(z-z^{\prime }\right)}^{2}}{\omega^{2}}\right)$$

(Section S2 in Data S1), where ω is the width of the competition kernel. The intrinsic growth $r_{0}\left(z\right)$ is 1 if z falls in the range $\left[-0.5,0.5\right]$ and 0 otherwise. This means that, although z can in principle take on any real value, positive growth is only possible in that range.

#### 2.1.2 Model 2: Resource‐Mediated Evolutionary HOIs (evoHOI)

**FIGURE 1:**
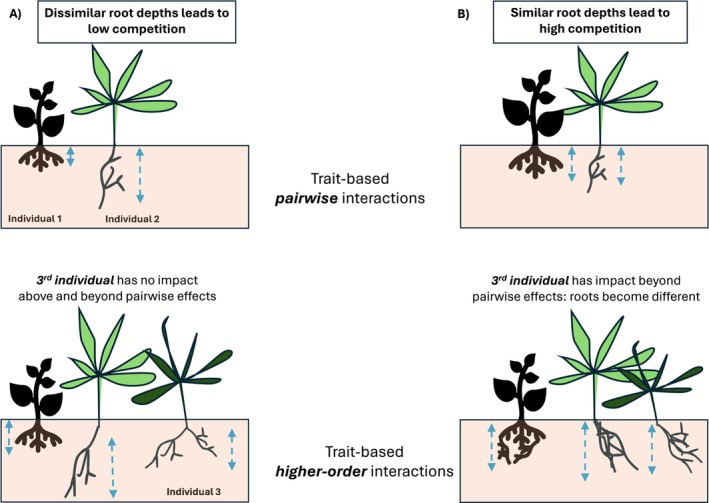
Example illustration of trait‐based pairwise interactions (top row) and trait‐based higher order interactions (bottom row). (A) When two nearby individuals have dissimilar root depths (blue dashed arrows), their low‐resource niche overlap reduces competition between them. Adding a third individual with a rooting depth that is distinct from both (bottom left), resource overlap and therefore competition remains low between all pairs. The impact of the third individual on any effect between the first two is thus also minimal. (B) When the root depths of two nearby individuals are similar, there is high‐resource use overlap between them which leads to intensified pairwise competition. In the presence of a third individual with similar root depth to the other two (bottom right), it is not just the three pairwise effects that intensify: Above and beyond that, each individual also experiences an extra reduction in performance. This is because squeezing three individuals in a confined space means that the only way for their roots to fit in the soil is to contort their morphology in a suboptimal manner. In this way, the presence of a third individual impacts how the other two perform, depending on their relative phenotypes (root depths).

This model is like evo above, except with trait‐based higher order terms included. The higher order kernel has the form

(5) $$W\left(z,z^{\prime },{z^{\prime }}^{\prime }\right)=\kappa \sqrt{\frac{4}{3\sqrt{\pi }\omega }}\exp\left(-\frac{{\left(z-z^{\prime }\right)}^{2}+{\left(z^{\prime }-{z^{\prime }}^{\prime }\right)}^{2}+{\left({z^{\prime }}^{\prime }-z\right)}^{2}}{3\omega^{2}/2}\right),$$

where κ scales the overall magnitude of the higher order effects, and ω is the same width parameter as in the pairwise kernel. This interaction term is a natural extension of the pairwise model, in terms of the resource utilization overlap of phenotype triplets that arise when three phenotypes could potentially interact along a trait axis (see Section S3 in Data S1 for the derivation). This interaction kernel is therefore high when the utilization functions of three phenotypes overlap, indicating strong higher order effects. The effect of scramble competition between three similar phenotypes is therefore not simply the sum of the separate scramble competitions across each pair, but additionally proportional to the three‐way overlap in resource utilization. Biologically, this could mean that three similar herbivore phenotypes competing for the same plant resources become wary of one another to the point where it disproportionately reduces their foraging efficiency compared with the pairwise scenario. Alternatively, one can think of root competition between plants. Two plants in a given location compete normally, by trying to utilize the resources in the soil better than their competitor. But when three plants occupy the same site and have similar root depths, there might no longer be sufficient space in the soil for all their roots—and so the plants must invest in twisting their roots into more compact shapes, reducing their uptake efficiency in the process (Figure 1). Both these scenarios would lead to negative three‐way effects.

#### 2.1.3 Model 3: Pairwise Hierarchical Model (hier)

Like for evo, in this model there are no HOIs, so $W\left(z,z^{\prime },{z^{\prime }}^{\prime }\right)=0$. Pairwise interactions are described by the hierarchical competition kernel

(6) $$a\left(z,z^{\prime }\right)=\frac{1}{2}\left[\mathrm{erf}\left(\frac{z-z^{\prime }}{\omega }\right)+1\right],$$

where $\mathrm{erf}\left(x\right)$ is the error function (Section S4 in Data S1). This kernel follows a sigmoid curve approaching 0 for $z\ll z^{\prime }$ and 1 for $z\gg z^{\prime }$. That is, individuals with a lower phenotype value are competitively superior to those with higher phenotype values. In line with classical competition‐mortality trade‐off models (Adler and Mosquera 2000), larger z values imply higher intrinsic growth rates but a lower rank in the hierarchy. Here, the intrinsic rates are implemented via $r_{0}\left(z\right)=1-\exp\left(-z\right)$. This implies that positive growth is only possible for z>0, even though z may in principle take on any positive real value.

#### 2.1.4 Model 4: Hierarchical Evolutionary HOIs (hierHOI)

This model is the same as hier above, but with trait‐mediated HOIs defined by the kernel

(7) $$W\left(z,z^{\prime },{z^{\prime }}^{\prime }\right)=\frac{\kappa }{2}\left[\mathrm{erf}\left(\frac{z_{0}+z-\left(z^{\prime }+{z^{\prime }}^{\prime }\right)/2}{\Omega }\right)+1\right]$$

(Section S5 in Data S1). In the previous model (hier), if an individual had a larger z, they were competitively inferior to another individual with a lower z. Now, with another phenotype ${z^{\prime }}^{\prime }$, the competition between the two phenotypes z, and $z^{\prime }$ depends on the position of the third phenotype ${z^{\prime }}^{\prime }$. It does so in a way that the competitive superiority of z is now evaluated over the average of $z^{\prime }$ and ${z^{\prime }}^{\prime }$, instead of either of those phenotypes individually. Biologically, this translates to a scenario where a superior competitor dominates an inferior one, but another competitor modulates this pairwise competition depending on its trait value or its position on the hierarchical trait axis (Abrams 2007; van Veen et al. 2005). Such an asymmetry is often observed in plant root competition in nutrient‐poor soil or plant competition for light (Rasmussen et al. 2019). Ω is the width of the sigmoid transition from 0 to 1, and $z_{0}$ is a constant modulating how much competitive superiority the third individual (with phenotype ${z^{\prime }}^{\prime }$) is capable of mitigating between z and $z^{\prime }$.

## 3 Numerical Simulations of the Models

### 3.1 Impact of Individual Variation and Trait‐Based HOIs on Species Coexistence

We assessed the effect of different levels of intraspecific trait variation on community structure and species coexistence using data generated from simulations of our community models. We simulated both trait dynamics and population dynamics resulting from Equations (2), (3). We started each simulation with 40 species at densities $N_{i}=1$, and with randomly assigned initial mean trait values (between −0.5 and 0.5 for evo and evoHOI; between 0 and 2 for hier and hierHOI). The intraspecific trait standard deviations $\sigma_{i}$ were sampled from one of three possible uniform distributions: either from low, medium, or high values (see Table 1 for these and other parameters). We also tested the influence of the breadth of pairwise competition, measured by the width of the pairwise competition kernel ω which could be either 0.1, 0.2, or 0.5.

**TABLE 1:** Parameters and their values with description used in the study. $U\left[a,b\right]$ is the uniform distribution between a and b.

Symbol	Description
$N_{i}$	Population density of species i. Their initial value is 1 for all species.
$u_{i}$	Mean trait value of species i. Initial values are sampled from $U\left[-0.5,0.5\right]$ for evo and evoHOI, and from $U\left[0,2\right]$ for hier and hierHOI.
$\sigma_{i}$	Trait standard deviation of species i; sampled from either $U\left[0.003,0.009\right]$ (low), $U\left[0.01,0.03\right]$ (medium), or $U\left[0.05,0.1\right]$ (high).
$h_{i}^{2}$	Heritability of species i's trait; sampled from $U\left[0.1,0.15\right]$.
θ	Width of intrinsic growth curve; 0.5 for evo/evoHOI and 1 for hier/hierHOI.
ω	Width of competition kernel for evo and evoHOI; either 0.1, 0.2, or 0.5.
κ	Relative magnitude of higher order terms; 10 for evoHOI and 5 for hierHOI.
Ω	Width of higher order interaction kernel for hierHOI, set to 0.01.
$z_{0}$	Shift parameter of higher order interaction kernel for hierHOI, set to 0.15.

**FIGURE 2:**
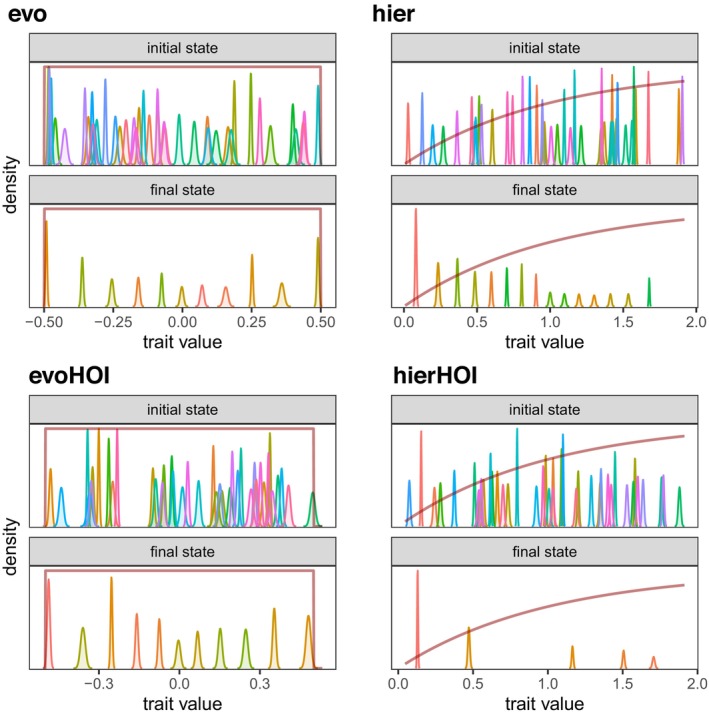
Eco‐evolutionary dynamics of many different species competing along a trait axis with four different types of species interactions: Evo, evoHOI (evolutionary HOIs), hier model (hierarchical pairwise), hierHOI model (hierarchical higher order). For each model, the top panel is the initial and the bottom panel the final, eco‐evolutionary equilibrium distribution of species' traits along the one‐dimensional trait axis. The red lines show the shape of the intrinsic growth rate function in each scenario (not drawn to scale). In each simulation, intraspecific trait standard deviations are low and ω=0.1; see Table 1 for all other parameters.

**FIGURE 3:**
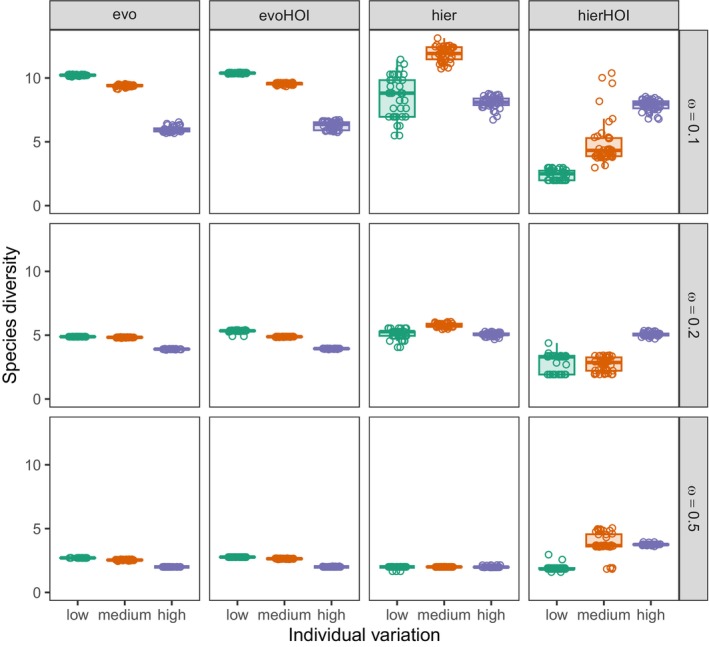
Effect of individual trait variation (*x*‐axis), different competition widths (rows), and model choice (columns) on species diversity (inverse Simpson's diversity index). Box plots summarize the number of species that coexisted at the end of 40 replicate simulations; points show the actual individual simulation results (they have been jittered sideways to reduce overlap). Trait‐based HOI models rarely lead to more species coexisting in comparison to pairwise models except when HOIs are hierarchical (hierHOI), the pairwise competition width is large (ω=0.5), and individual variation is not too low.

**FIGURE 4:**
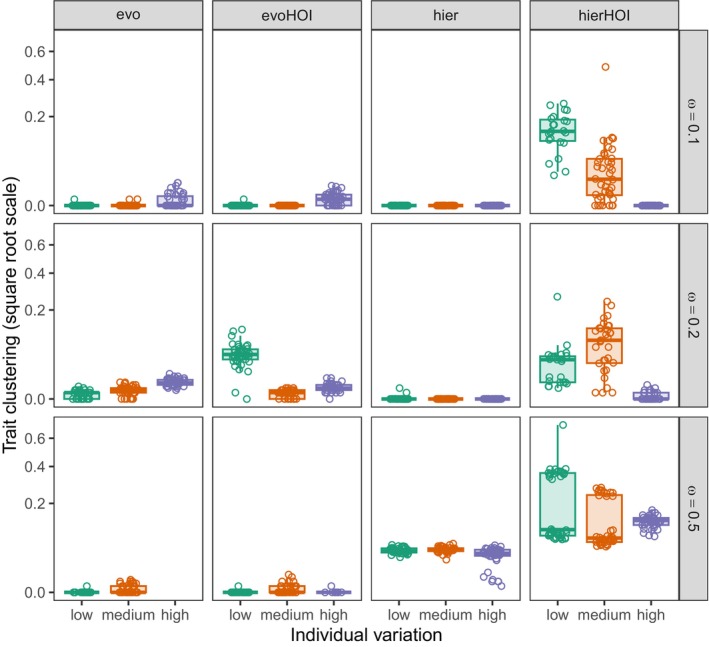
As Figure 3, but with trait clustering along the *y*‐axis. A score close to 0 means the community's species are much more evenly spaced than expected by chance, while a score close to 1 means the opposite: The species are more clustered than expected. Pairwise models (evo, hier) and HOIs (evoHOI, hierHOI) all lead to very even trait spacing, although hierHOI can lead to somewhat higher clustering. The data are missing for the evo model at ω=0.5 and high individual variation because in that scenario, it was always two species surviving in all 40 replicates, so there is no meaningful measure of trait clustering in that case.

**FIGURE 5:**
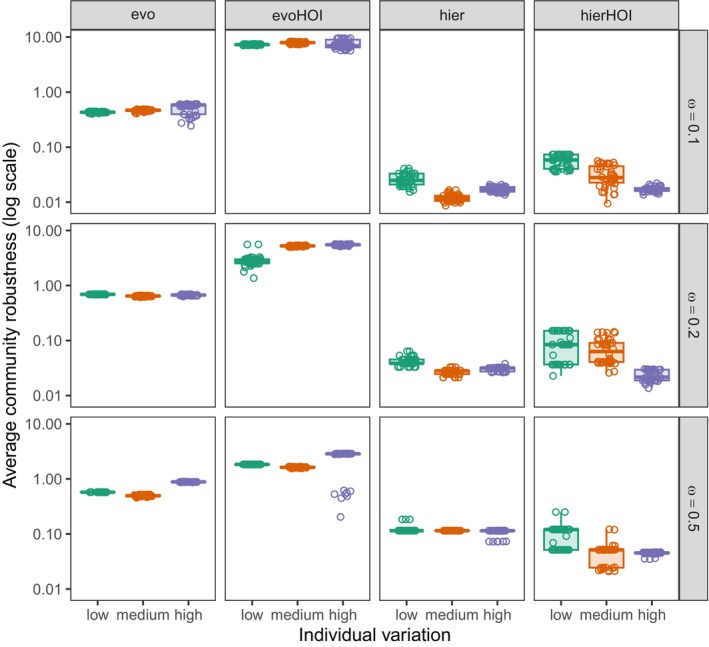
As Figure 3, but with average community robustness along the *y*‐axis (on the log scale). Higher values denote higher community robustness against environmental perturbations.

The four models, together with three possible levels of intraspecific variation and three possible values of ω, result in 36 combinations. For each of these, we carried out 40 replicate simulations where we integrated the community dynamics until they reached an eco‐evolutionary equilibrium (this was always the observed outcome; in no cases did we see eco‐evolutionary cycles or chaos emerge). These 40 replicates were sufficient to detect patterns of interest: in Figures 3, 4, 5, the data points in the box plots cluster similarly, indicating consistent patterns across all scenarios (see Results section). Species diversity was then quantified using the inverse Simpson index $D=1/\sum_{i}\;p_{i}^{2}$, where $p_{i}=N_{i}/\sum_{i}\;N_{i}$ is the proportion of the community made up by species i. Figure 2 illustrates one simulated outcome from each of the four models.

We took care to use the same initial trait means in corresponding replicates across models. For example, replicate 1 for both evo and evoHOI under low individual variation and ω=0.1 has the same, pseudorandomly generated initial trait means. This keeps replicates of different models comparable. The same holds for individual variation: while the $\sigma_{i}$ differed across replicates, they were equal within a replicate even if the other factors were altered.

### 3.2 Trait Clustering

Here, we use a quantitative metric to evaluate the effect of intraspecific variation on the patterning of traits in the trait axis. We measured trait similarity among coexisting species with the coefficient of variation (CV) of adjacent trait means (D'Andrea and Ostling 2016). To evaluate whether the observed trait clustering was greater than expected by chance, we used a null model consisting of trait values of species drawn from a uniform distribution matching the final species richness and limits. The trait values of the final surviving species were linearly transformed by replacing every $u_{i}$ with $\left(u_{i}-\min\left(u_{i}\right)\right)/\left(\max\left(u_{i}\right)-\min\left(u_{i}\right)\right)$. This makes the lowest surviving mean trait 0, the highest 1, and adjusts everything proportionally in between. We then compared the CV of the observed community with 1000 corresponding CVs from the null model and tallied the fraction of null CVs that were lower than that of the observed community. This *p* value can then be used to quantify trait clustering: low (high) *p* values indicate that the species in the community are more evenly spaced (more clustered) along the trait axis than expected by chance.

### 3.3 Robustness of Species Coexistence

At the end of our simulations, we measured the (ecological) robustness of a community by first calculating the community matrix (Jacobian of the ecological part of the dynamics, evaluated at equilibrium; Section S8 in Data S1) and obtaining its eigenvalues. Robustness is then measured by taking the geometric mean of these eigenvalues' absolute values (Barabas and D'Andrea 2016). For S species, this is the same as the Sth root of the absolute value of the matrix's determinant. It measures the (geometric) average return times of the system to equilibrium after a perturbation of the population densities, and also quantifies the system's sensitivity against parameter perturbations (Barabás et al. 2014).

## 4 Results

### 4.1 Trait‐Based HOIs and Individual Variation on Species Diversity

For the resource‐mediated competition models (evo and evoHOI), both increasing intraspecific variation and increasing the competition width lead to a decrease in species diversity (Figure 3). This result is consistent with prior work for the pairwise (evo) model and makes intuitive sense: Wider competition kernels lead to larger gaps between coexisting species, and broader trait distributions lead to species utilizing a broader spectrum of resources in this model, yet again leading to more sporadically spaced competitors (Barabas and D'Andrea 2016). Perhaps disappointingly, higher order interactions do not affect this outcome: The observed species diversities in corresponding parameterizations for the evo and evoHOI models are essentially indistinguishable in all cases.

This changes for the trait‐based hierarchical models. In the pairwise (hier) case, there is again an overall decrease of species diversity with ω. However, the effect of individual variation is less clear‐cut. As seen in Figure 3, going from low to intermediate levels of intraspecific trait variance leads to an increase in richness. But increasing individual variation further results in a sharp drop instead, so there is still an overall negative effect of large individual variation on the number of coexisting species. Adding higher order interactions (hierHOI) changes the corresponding pairwise diversities in all three conceivable ways. Namely, when neither ω nor intraspecific variability are too large (ω=0.1 or 0.2, and individual variation is either low or medium), there is a drop in diversity. For large individual variation and not too large ω, diversity is unaffected. However, for large ω and medium to high individual variation, higher order interactions increase diversity compared with the pairwise (hier) model.

Overall, trait‐based HOIs often have a negligible or negative impact on species diversity, with some notable exceptions where HOIs are beneficial. In Section 4.4 we outline a framework for making sense of these results. We argue that while the outcomes appear confusing on the surface, it is possible to gain an understanding of the influence of higher order interactions in any particular scenario by analyzing the widths of the pairwise and higher order interaction kernels.

### 4.2 Trait‐Based HOIs and Individual Variation on Trait Clustering

Overall, in all but a few outlying cases, traits are more evenly spaced than expected by chance, and often substantially so (Figure 4; note that the y‐axis is on a square root scale). This confirms the generality of the patterns seen in Figure 2. Notably, species in the hierHOI model tend to be less evenly spaced than in the other three, except when individual trait variation is high and the pairwise competition width is not too large (ω<0.5). There is a broad overall trend that simultaneously increasing individual variation and the pairwise competition width leads to more trait clustering. However, this tendency is not very strong, and sometimes violated at smaller scales; for example, for the hierHOI model with small ω, the relationship is reversed.

We do observe much higher degrees of trait clustering as well, but only when HOIs are parameterized not by traits but random numbers and by certain rules such as intraspecific HOI effects being stronger than interspecific HOI effects (intraHOI; Section S7 in Data S1). This specific structure of HOIs also points toward the fact that such higher order effects are mediated by either environmental effects or other traits that are not related to the competitive one‐dimensional trait that we model. As the higher order interaction structure is random in these models, its lack of a definite trait clustering is understandable. Why does intraHOI model produce such highly clustered patterns? The reason is that in this model, intraspecific effects are stronger than interspecific ones by assumption. This extra burden of prescribed self‐regulation means that species cannot easily exclude one another even when they are very close on the trait axis governing the pairwise interactions. Thus, multiple species can converge into local evolutionary optima and coexist there (Figures S1 and S2).

### 4.3 Trait‐Based HOIs and Individual Variation on Robustness of Species Coexistence

The largest robustness is associated with the evo and evoHOI models, where species are evenly spaced and their interactions decrease with trait distance (Figure 5). Interestingly, adding higher order effects to this model boosts robustness, even though it does not affect species diversity in any way (Figure 3). This is likely a result of the fact that the higher order terms in this model are stronger for more similar phenotypes—therefore, in a community with just as many evenly spaced species as in the pairwise case, each species experiences an additional self‐regulatory term, which makes species even more independent and therefore robust to external perturbations. In the hierarchical models where a species higher in the hierarchy competitively affects every species below it (in contrast with the evo and evoHOI models where a species mostly interacts with its direct neighbors), robustness is consistently lower than in the resource overlap models. Adding higher order effects (hierHOI) does not affect these robustness values much.

### 4.4 Analytical Argument on How Trait‐Based HOIs Affect Coexistence

To evaluate the expected effect of trait‐based HOIs on species diversity, we start from a known fact about pairwise models: The average spacing between species along the trait axis is proportional to the width of the interaction kernel (MacArthur and Levins 1967; Szabó and Meszéna 2006; Barabás and Meszéna 2009; Barabas and D'Andrea 2016). This holds regardless of model details, which means that the same principle applies in both the evo and hier models (Barabás et al. 2012; D'Andrea et al. 2013). If only a fixed portion of the trait axis is filled with species, this also means that species diversity is roughly inversely proportional to the kernel width. More precisely, it will be proportional to the effective width that arises after accounting for the intraspecific variability of the species, which always acts to broaden the kernel's width (Barabas and D'Andrea 2016; Section S9 in Data S1).

As discussed in Section S1.3 in Data S1, the HOI model can be thought of as a pairwise model with an effective interaction kernel that is the sum of the true pairwise effects and the higher order effects summed over all third participants in the three‐way interaction:

(8) $$\alpha^{\mathrm{eff}}\left(u,u^{\prime }\right)=\alpha \left(u,u^{\prime }\right)+\int \varepsilon \left(u,u^{\prime },{u^{\prime }}^{\prime }\right)N\left({u^{\prime }}^{\prime }\right)\;d{u^{\prime }}^{\prime }.$$

Here, $\alpha \left(u,u^{\prime }\right)$ and $\varepsilon \left(u,u^{\prime },{u^{\prime }}^{\prime }\right)$ are analogous to $a\left(u,u^{\prime }\right)$ and $W\left(u,u^{\prime },{u^{\prime }}^{\prime }\right)$ from Equation (1), except they have already been integrated across the trait distributions of the species with mean trait values u and $u^{\prime }$, respectively—that is, they already account for individual variation in traits. $N\left(u\right)$ is the distribution of population densities as a function of the species mean trait u. While the shape of the integrated part cannot be computed without knowing the final density distribution $N\left(u\right)$, we can assume as a crude first approximation that its shape matches $\varepsilon \left(u,u^{\prime },{u^{\prime }}^{\prime }\right)$ (but with the ${u^{\prime }}^{\prime }$ variable integrated out). Its width is thus also proportional to that of $\varepsilon \left(u,u^{\prime },{u^{\prime }}^{\prime }\right)$. Given this approximation, the shape of the effective kernel $\alpha^{\mathrm{eff}}\left(u,u^{\prime }\right)$ arises from the sum of the shapes of the pairwise and higher order contributions Equation (8).

But then, since in the pairwise case we know that the kernel's width is the main determinant of species richness, and the effective kernel formally reduces the HOI model to a pairwise one, we can conclude that in HOI models, the width of the effective kernel governs the average spacing between adjacent species. This is our main insight: *to think about coexistence in a HOI model, one should think of it as if it were a pairwise model, but with an effective interaction kernel whose shape follows the sum of the pairwise and HOI contributions.*

The overall width of such a sum of kernel shapes generally matches the width of the broader of the two functions. Thus, in case the HOI kernel is narrower than the pairwise one, it will still be the pairwise kernel dictating the overall spacing between species—diversity is therefore unaffected by the addition of HOIs (Section S10: Scenario 1 in Data S1). Conversely, if the HOI kernel is wider than the pairwise one, then we expect diversity to decline after adding it because the width of the effective kernel will now match that of the HOI contribution (Section S10: Scenario 2 in Data S1). The typical outcomes are therefore either no effect or a negative effect of HOIs on diversity.

However, there may be exceptions to this whenever the HOI kernel's width is either extremely large or extremely small. First, if the HOI kernel width is very large, then HOI interactions between any two phenotypes are essentially equal. It then contributes a constant increase to the effective kernel and does not change its width (Section S10: Scenario 3 in Data S1). In that case, instead of making the spacing between species proportional to the width of the HOI kernel, it will yet again become proportional to that of the pairwise one. That is, one gets more diversity than if the HOI kernel had been narrower. Second, when the HOI kernel width is very small without the pairwise one being too extreme, this can introduce an effect whereby the larger width separates clusters of species, but within a cluster it is the smaller width determining the spacing (Barabás et al. 2013). In such situations, adding HOIs will actually promote diversity compared to the pairwise case (Section S10: Scenario 4 in Data S1).

## 5 Discussion

Our study examined a trait that influences competitive ability and incorporated higher order interactions involving a third species. We found that increasing intraspecific trait variation leads to a reduction in species diversity, consistent with findings by Barabas and D'Andrea (2016) (Figure 3). When trait‐based HOIs are introduced, they mostly have either a negative or no effect on diversity. However, we argue that in these models, species coexistence by higher order effects are primarily governed by the relative widths of the pairwise and HOI competition kernels. This understanding allows one to construct scenarios where HOIs are beneficial for diversity which we observe when trait‐based hierarchical HOIs were at play, but not when distance‐alleviated trait‐based HOIs “evoHOI” were at play (e.g., Figure 3, hierHOI, ω=0.5 with medium or high individual variation).

We reasoned about the effect of trait‐based HOIs by translating HOI scenarios to effective pairwise models whose interaction kernel is the sum of the original pairwise and HOI kernels. While this way of thinking does offer a useful perspective, it is nevertheless important to keep in mind that this is just a heuristic. It cannot hold in a quantitatively precise way because Equation (8) is not simply the sum of the pairwise and higher order contributions. Instead, the HOI terms are themselves added up over all third species. This sum will not have a well‐defined width that is independent of trait position, so one can only hope that it remains sufficiently constant not to invalidate our logic. For this reason, one should not expect predictions based on the summed kernel to be quantitatively correct, and finding a fully accurate way of predicting the effects of trait‐based HOIs still requires further research. That said, thinking in terms of the summed kernels does appear to work well qualitatively (Section S10 in Data S1). Indeed, this was the reason why we found scenarios in which higher order interactions promoted diversity in the first place. While initially we only saw examples that either have no effect or are detrimental, once we realized that it is possible to think of HOI models as effective pairwise ones with summed kernels, it was easy to create situations where HOIs are beneficial to diversity.

Whether such combinations are artificial constructs or have a chance of arising in nature remains to be observed. However, one could argue that they could potentially occur under certain environmental conditions. Indeed, under experimental scenarios, it has been suggested that the strength and the presence of HOIs could be environmentally dependent (Fox 2023), which consequently can impact species coexistence. Without such effects, given the fact that HOIs only promoted diversity in small and specially chosen regions of parameter space, the a priori likelihood of this happening appears slim. This is especially the case with models like our evoHOI, because in that model both the pairwise and higher order kernel widths arise from the underlying resource utilization function. Their interrelatedness will therefore make it impossible to change the kernel widths independently, and so the diversity‐promoting combination in Section S10: Scenario 4 in Data S1, is not achievable without some other model ingredient that could independently reduce the width of the higher order kernel only.

Our results suggest that with the introduction of three‐way interactions, the dominance of a competitively superior species and its impact on coexistence is rarely dependent on the structure and the manner in which HOIs impact pairwise competition. Specifically, in pairwise competing species, when a third species strengthens intraspecific competition more than interspecific competition (intraHOI; Figure S1), species coexistence is promoted, especially at lower levels of competition width. This is because the third species further restricts the growth of the other two species by increasing intraspecific pairwise competition (Singh and Baruah 2021). This is analogous to the pairwise coexistence rule where species must limit themselves more than limit others in order for coexistence to prevail (Figure 3). Such HOIs could be considered high‐dimensional as these HOIs are random coefficients sampled from a wider distribution than HOIs emerging from constraints imposed by species mean trait values. Such higher order effects could be a manifestation of a variety of underlying unmeasured traits of species or could be a result of variation in environmental conditions (Fox 2023). Sampling specific random HOIs thus could fairly mimic such a scenario. Trait‐based HOIs as modeled in evoHOI further disrupted species coexistence or had little impact on species coexistence in comparison with pairwise species competition. In the pairwise model, two species with similar phenotypic trait values experience intense competition, as described by the pairwise Gaussian competition kernel. If a third species in the evoHOI model also shares similar phenotypic traits, this further amplifies both intra‐ and interspecific competition. As a result, this has a greater impact on overall species diversity (Figure 2, evoHOI), regardless of the width of species competition.

Hierarchical communities such as those in our hier and hierHOI models have a specific structure of species competition. Competition is not only dependent on one's neighbors along the trait axis, but dependent on the entire trait axis. For instance, a competitor with a lower trait value will impose its dominance in terms of competition to all the species below that trait value. Such hierarchical communities thus are different than the evo and evoHOI communities. With this framework, in the hier model, high trait variation still leads to low species diversity. By contrast, in hierHOI, species coexistence was promoted only at the highest level of ω=0.5 and at higher levels of trait variation. Why do we observe higher number of species coexisting at higher ω=0.5 and at high levels of trait variation (Figure 3)? This can be explained by examining the hierHOI and hier kernels, and how these together influence the total competitive effect faced by a competitively weaker species. In Figure S4 and Section S10 in Data S1, we heuristically demonstrate how effective competition can be modulated by the relative widths of pairwise and HOI kernels. We demonstrated that at the highest level of hierarchical pairwise competition $\left(\omega =0.5\right)$, species with no trait variation experience a markedly different total effective competitive effect compared to those with positive trait variation. Specifically, the slope of the total competitive effect as well as the strength is slightly reduced as trait differences increase in comparison to the case when there is no individual variation. As a consequence, due to lesser slope when trait variance was high and due to less strong of a effective competitive effect, a slightly higher number of species coexist when hierHOI was at play and the spacing between species then was dictated by the slope leading to slightly even spacing as we observe in Figure 4. Thus, at $\left(\omega =0.5\right)$ and with positive trait variation, weaker competitors experienced less total effective competition compared to scenarios where trait variation was absent (Figure S3). As a result, hierHOI here, markedly alleviates competition only when species trait variation is high which results in slightly increased species competition which we observe in Figure 3 at a high ω=0.5.

Trait variation within species in a community is widely observed, but the implication of such variation on the patterning of traits is still debated (Götzenberger et al. 2012). In our eco‐evolutionary model, where competition between species included both pairwise and trait‐mediated HOIs, increases in trait variation led to low trait clustering and more even spacing (Figure 4). When there is high heritable variation in the trait, evolution would be faster compared to when there is low heritable variation. Thus, as expected due to faster evolutionary dynamics caused by high heritable variation, species would move away from each other leading to lesser trait overlap and greater trait divergence in comparison to when heritable variation is low. When evolutionary HOIs came into play, trait divergence due to strong pairwise competition was further exacerbated particularly when all the three species involved in HOIs had similar mean phenotypic traits. Evolutionary HOIs, thus, further reinforced the limiting similarity principle irrespective of the presence of high or low individual variation. Higher trait patterning, however, was seen only when intraspecific random HOIs were modeled to be stronger than interspecific random HOIs (Figures S1 and S2). This specific random structure of HOIs essentially just increases self‐regulation or strengthens intraspecific competition in a way that allows for high species clustering without any competitive exclusion. In contrast, trait clustering in hierarchical models were slightly unique than all the other models compared. Despite that high trait variation still led to low species clustering in the trait axis.

Recent advances in understanding community stability with eco‐evolutionary dynamics reveal that multispecies communities can be stable or unstable based on the relative speed of ecological and evolutionary processes (Patel et al. 2018). Our study indicates that higher intraspecific variation can lead to robust species coexistence in the presence of HOIs (Figure 5). Increased intraspecific variation accelerates evolutionary dynamics, favoring coexisting species with advantageous positions along a one‐dimensional trait axis. However, this effect may be due to a lower number of coexisting species, enhancing average community robustness. Pairwise and higher order hierarchical models show relatively low community robustness, and slight perturbations could destabilize the coexistence equilibrium, resulting in the extinction of competitively inferior species, as from Figure 3, these species are at very low density.

Throughout this work, we assumed strictly competitive effects between individuals. The extension of our modeling framework to noncompetitive interactions is in principle straightforward: All one needs to do is specify pairwise and higher order interaction kernels $a\left(z,z^{\prime }\right)$ and $W\left(z,z^{\prime },{z^{\prime }}^{\prime }\right)$ in Equation (1) that are not strictly positive. This could be the case, for example, if the modeled trait is body size, and trophic interactions always happen between individuals with a given body size ratio (Schneider et al. 2016). That said, two things are worth keeping in mind. First, Equation (1) could in principle produce mathematical blow‐up when the kernels are not positive—this should either be avoided via proper parameterization, or by changing the model's structure. Second, the assumption that species differ only in a single trait already predisposes the model's applicability toward species that are similar to one another. But sufficiently similar species necessarily compete. However, if in our theoretical model, very similar phenotypes had a net positive *inter*specific effect, they would theoretically also exert the same effect *intra*specifically as well which then would lead to unchecked population growth. For these reasons, while extending the framework to noncompetitive interactions is possible, it is natural to apply it to competitive situations in particular.

Our results show that, across various competitive communities, low‐dimensional higher order effects have an easy time disrupting coexistence, but have a hard time promoting it. This suggests that for HOIs to promote coexistence, they must generally be high‐dimensional or emerge under specific environmental conditions (Fox 2023), or act in scenarios where higher order effects act on a narrow trait distance relative to where pairwise competition comes into play. Recent studies have similarly indicated that even random sampling of HOIs (high‐dimensional HOIs) may still not be enough to promote species coexistence (Gibbs et al. 2024). This highlights the need for precise modeling of HOIs, incorporating multidimensional traits and mechanisms distinct from those governing pairwise competition to see whether and when (if at all) higher order interactions contribute to observed diversity patterns.

## Author Contributions

**Gaurav Baruah:** conceptualization (equal), data curation (equal), formal analysis (equal), funding acquisition (equal), investigation (equal), methodology (equal), resources (equal), software (equal), validation (equal), visualization (equal), writing – original draft (equal), writing – review and editing (equal). **György Barabás:** conceptualization (equal), data curation (equal), formal analysis (equal), investigation (equal), methodology (equal), project administration (equal), resources (equal), software (equal), validation (equal), visualization (equal), writing – original draft (equal), writing – review and editing (equal). **Robert John:** conceptualization (equal), methodology (equal), resources (equal), supervision (equal), validation (equal), writing – review and editing (equal).

## Conflicts of Interest

The authors declare no conflicts of interest.

## Supporting information


Figure S1.
Figure S2.
Figure S3.
Figure S4.

Data S1.

## Data Availability

Code, data, and figures are available from https://github.com/dysordys/trait‐based‐hoi.
