# Supplementary material for: When Do Trait‐Based Higher Order Interactions and Individual Variation Promote Robust Species Coexistence?
Source: Ecol Evol. 2025 Apr 25;15(4):e71336. doi: 10.1002/ece3.71336 (PMC12031895; doi:10.1002/ece3.71336)
Supplement: Supplementary file 1 — Figure S1. Figure S2. Figure S3. Figure S4. [file ECE3-15-e71336-s002.pdf]

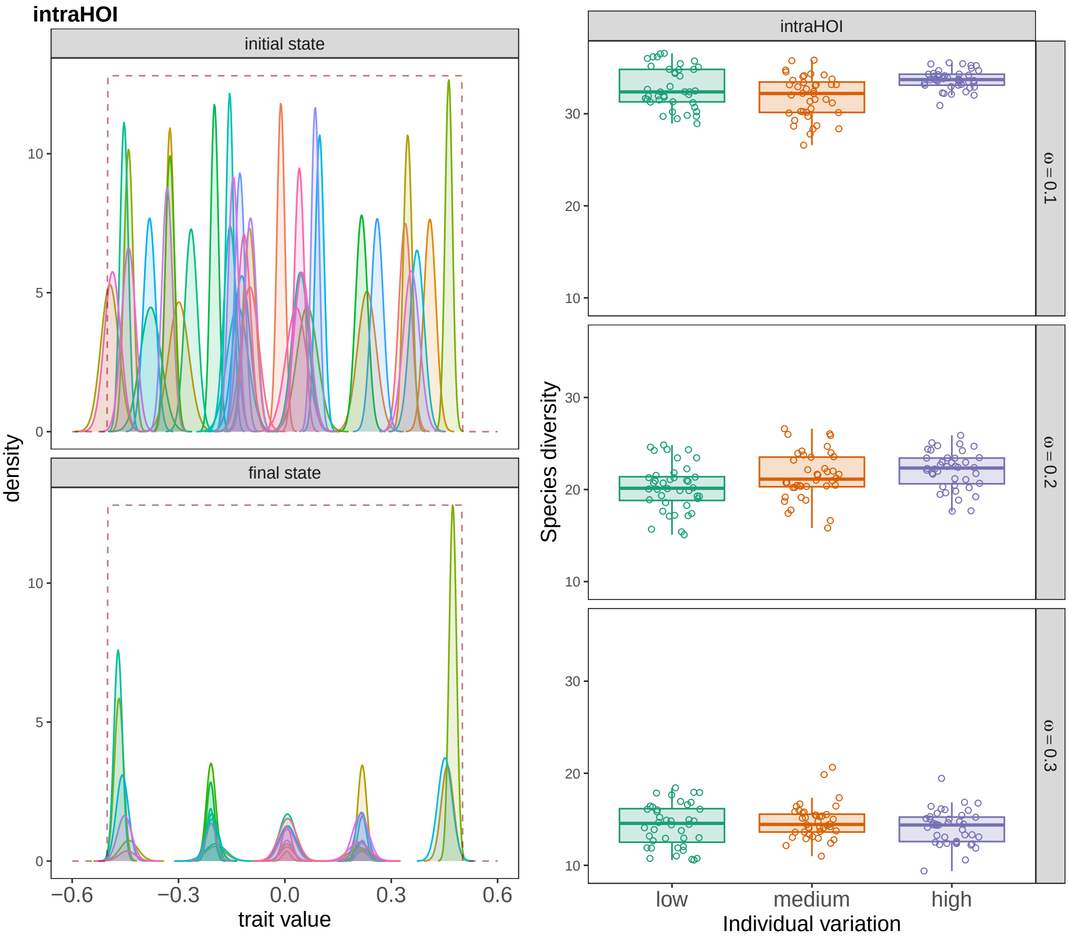

Figure S1: Species diversity (measured by the inverse Simpson index) in the intraHOI model where HOI coefficients were randomly generated by a specific structure such that intraspecific HOIs were greater in strength than interspecific HOIs. We observe trait clustering and higher diversity across different levels of ω.

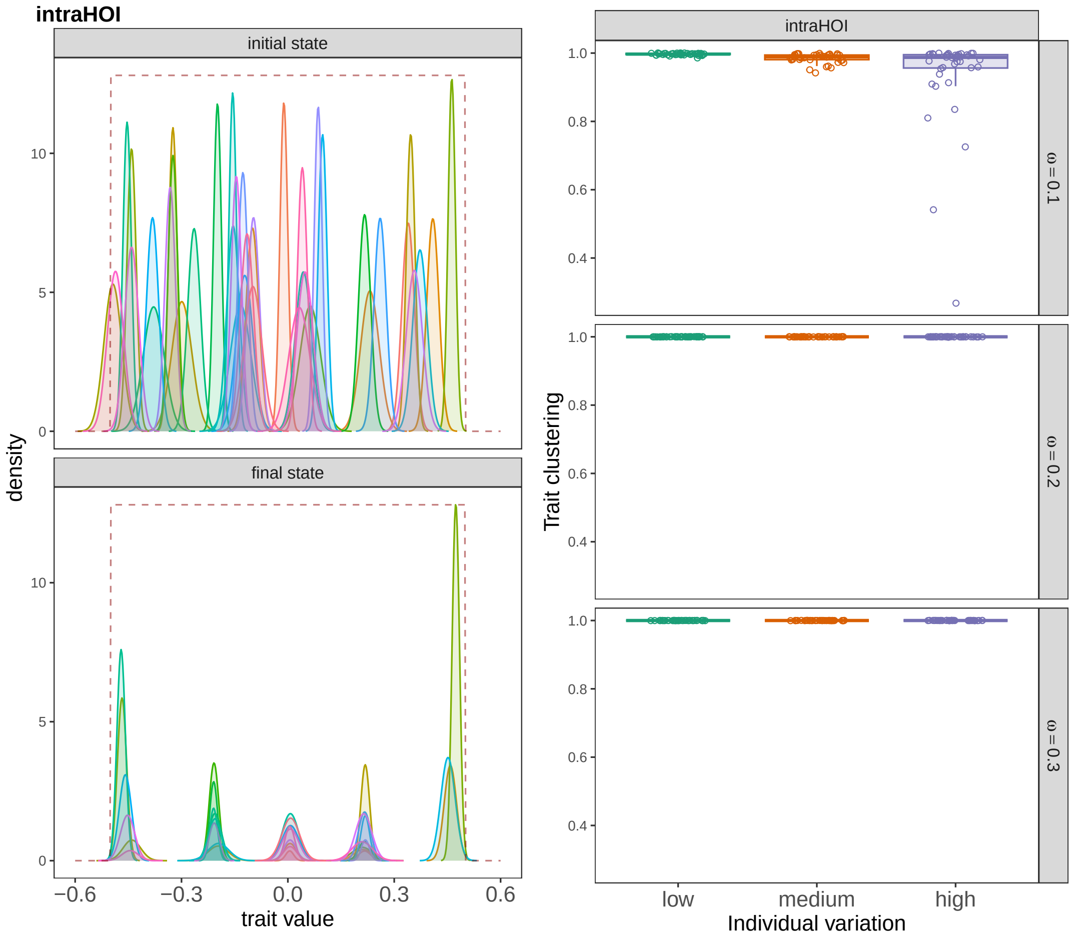

Figure S2: Species clustering in intraHOI model where HOI coefficients were randomly generated by a specific structure such that intraspecific HOIs were greater in strength than interspecific HOIs. In that case we do observe high amount of trait clustering across different levels of ω and three different levels of individual variation.

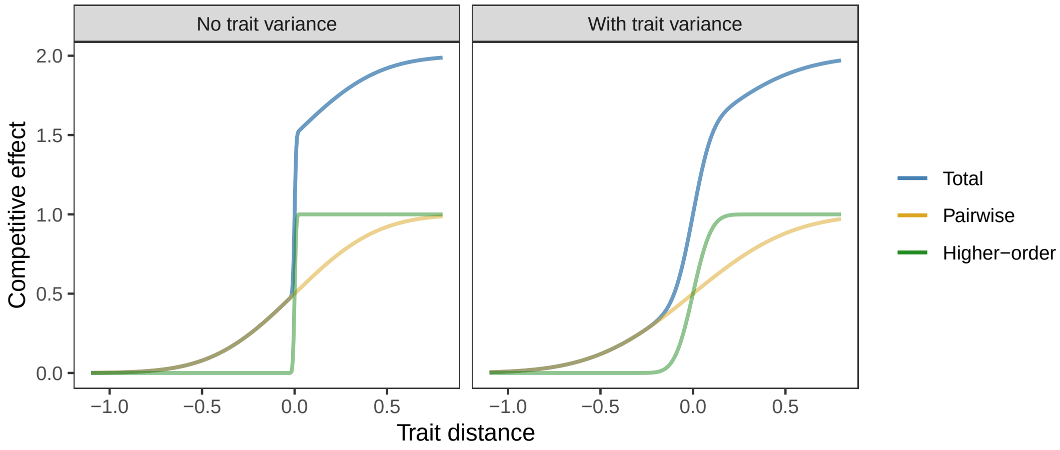

Figure S3: Competitive effect faced by species in the hierHOI model as a function of trait distance, with $\sigma_i^2 = 0$ (left panel) and $\sigma_i^2 = 0.1$ (right panel). The curves show the shape of the pairwise part of the interaction kernel (yellow), the higher-order part (green), and their sum (blue). Intraspecific variation smooths out both the pairwise and higher-order kernels—and as a consequence, their sum as well. Other parameters: $\omega = 0.5$, $\Omega = 0.01$, $\kappa = 1$.

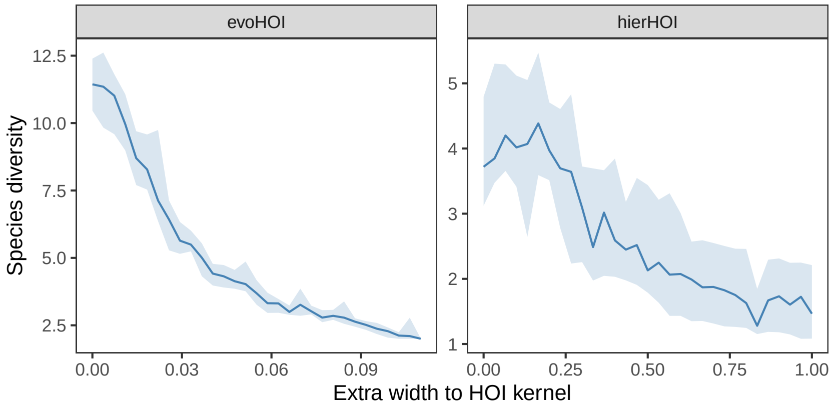

Figure S4: Increasing the widths of evoHOI (left) and hierHOI (right) higher-order kernels, and their impact on overall species diversity (measured by the inverse Simpson index). Here, pairwise interactions and evolutionary dynamics are eliminated by setting the pairwise kernel $a(z, z')$ and the heritabilities h_i^2 to zero. All other parameters are as in Table 1 from the main text (with low individual variation).
