# Supplementary material for: When Do Trait‐Based Higher Order Interactions and Individual Variation Promote Robust Species Coexistence?
Source: Ecol Evol. 2025 Apr 25;15(4):e71336. doi: 10.1002/ece3.71336 (PMC12031895; doi:10.1002/ece3.71336)
Supplement: Supplementary file 2 — Data S1. [file ECE3-15-e71336-s001.pdf]

Supplementary: When do trait-based higher-order interactions and individual variation promote robust species coexistence?

Gaurav Baruah[‡,1,*], György Barabás[‡,2,3], Robert John[4]
[1]Faculty of Biology, Theoretical Biology, University of Bielefeld, Bielefeld, 33501, Germany
[2]Division of Biology, Linköping University, Linköping, Sweden
[3]Institute of Evolution, Centre for Ecological Research, Budapest, Hungary
[4]Department of Biological Sciences, IISER Kolkata, Mohanpur, 741246, India

S1 General framework

S1.1 Per capita growth rates and governing equations

We model the eco-evolutionary dynamics of S interacting species, each composed of phenotypes z along a one-dimensional trait axis. The distribution $p_i(z)$ of the quantitative trait is Gaussian for each species i, with mean u_i and standard deviation σ_i:

$$p_i(z) = \frac{1}{\sqrt{2\pi}\sigma_i} \exp\left(-\frac{(z-u_i)^2}{2\sigma_i^2}\right). \tag{S1}$$

If N_i is the number of individuals of species i, then $N_i p_i(z)\,\mathrm{d}z$ is the population density of species i's individuals with trait value between z and $z+\mathrm{d}z$ (Lande 2009).

In the quantitative genetic approximation, many independent loci contribute to the phenotype z of any individual, each with a small additive effect. Under this model, the trait variances do not evolve, and the dynamics of the population densities N_i and the trait means u_i are given by the following pair of equations (Barabás & D'Andrea 2016, Pastore *et al.* 2021, Åkesson *et al.* 2021, Barabás *et al.* 2022):

$$\frac{\mathrm{d}N_i}{\mathrm{d}t} = N_i \int r(z) p_i(z)\,\mathrm{d}z, \tag{S2}$$

$$\frac{\mathrm{d}u_i}{\mathrm{d}t} = h_i^2 \int (z-u_i) r(z) p_i(z)\,\mathrm{d}z, \tag{S3}$$

where $r_i(z)$ is the per capita growth rate of species i's phenotype z, h_i^2 is the heritability of the trait z for species i, and all integrals go from $-\infty$ to $+\infty$.

The form of the per capita growth rates is determined by ecological interactions, and is in principle arbitrary. In this work, all models will have the following structure:

$$r(z) = r_0(z) - \sum_{j=1}^{S} N_j \int a(z,z') p_j(z')\,\mathrm{d}z' - \sum_{j=1}^{S}\sum_{k=1}^{S} N_j N_k \iint W(z,z',z'') p_j(z') p_k(z'')\,\mathrm{d}z'\,\mathrm{d}z''. \tag{S4}$$

Here $r_0(z)$ is the intrinsic growth rate of phenotype z, $a(z,z')$ is the pairwise phenotype-to-phenotype effect of z' on z, and $W(z,z',z'')$ is the three-way higher-order interaction (HOI) effect of z' on z given the presence of phenotype z''. We can substitute this expression into Eqs. S2-S3:

$$\begin{aligned}\frac{\mathrm{d}N_i}{\mathrm{d}t} = N_i \int \Bigg[& r_0(z) - \sum_{j=1}^{S} N_j \int a(z,z') p_j(z')\,\mathrm{d}z' \\ & - \sum_{j=1}^{S}\sum_{k=1}^{S} N_j N_k \iint W(z,z',z'') p_j(z') p_k(z'')\,\mathrm{d}z'\,\mathrm{d}z'' \Bigg] p_i(z)\,\mathrm{d}z,\end{aligned} \tag{S5}$$

$$\frac{\mathrm{d}u_i}{\mathrm{d}t} = h_i^2 \int (z-u_i) \Bigg[r_0(z) - \sum_{j=1}^{S} N_j \int a(z,z') p_j(z') \,\mathrm{d}z' \\ - \sum_{j=1}^{S}\sum_{k=1}^{S} N_j N_k \iint W(z,z',z'') p_j(z') p_k(z'') \,\mathrm{d}z' \,\mathrm{d}z'' \Bigg] p_i(z) \,\mathrm{d}z, \tag{S6}$$

and introduce some simplifying notation that will reveal the general structure of these equations:

$$b_i = \int r_0(z) p_i(z) \,\mathrm{d}z, \tag{S7}$$

$$\alpha_{ij} = \iint a(z,z') p_i(z) p_j(z') \,\mathrm{d}z' \mathrm{d}z, \tag{S8}$$

$$\epsilon_{ijk} = \iiint W(z,z',z'') p_i(z) p_j(z') p_k(z'') \,\mathrm{d}z'' \mathrm{d}z' \mathrm{d}z, \tag{S9}$$

$$g_i = \int (z-u_i) r_0(z) p_i(z) \,\mathrm{d}z, \tag{S10}$$

$$\beta_{ij} = \iint (z-u_i) a(z,z') p_i(z) p_j(z') \,\mathrm{d}z' \mathrm{d}z, \tag{S11}$$

$$\gamma_{ijk} = \iiint (z-u_i) W(z,z',z'') p_i(z) p_j(z') p_k(z'') \,\mathrm{d}z'' \mathrm{d}z' \mathrm{d}z. \tag{S12}$$

Using these quantities, we rewrite Eqs. S5-S6 as

$$\frac{\mathrm{d}N_i}{\mathrm{d}t} = N_i \left[b_i - \sum_{j=1}^{S} \alpha_{ij} N_j - \sum_{j=1}^{S}\sum_{k=1}^{S} \epsilon_{ijk} N_j N_k \right], \tag{S13}$$

$$\frac{\mathrm{d}u_i}{\mathrm{d}t} = h_i^2 \left[g_i - \sum_{j=1}^{S} \beta_{ij} N_j - \sum_{j=1}^{S}\sum_{k=1}^{S} \gamma_{ijk} N_j N_k \right]. \tag{S14}$$

These are the species-level equations for the eco-evolutionary dynamics. When $\epsilon_{ijk} = 0$, Eq. S13 reduces to a classic generalized Lotka–Volterra system with intrinsic rates b_i and interaction coefficients α_{ij}.

S1.2 Avoiding self-effects in higher-order interactions

The most principled way of modeling HOIs is to make sure that $W(z,z,z) = 0$. If this property does not hold, then we end up with "higher-order self-effects", a concept that is an oxymoron. Here we show that despite this fact, it is sufficient if $W(z,z,z) = W_0$ is a z-independent constant. The reason is that we can then redefine the higher-order kernel as $W'(z,z',z'') = W(z,z',z'') - W_0$, and in return add the common density-dependent factor $W_0(\sum_j N_j)^2$ to the per capita growth rates. Writing out only the HOI term of Eq. S4 (since the other terms are unchanged):

$$\begin{aligned}
&\sum_{j=1}^{S}\sum_{k=1}^{S} N_j N_k \iint W'(z,z',z'') p_j(z') p_k(z'') \,\mathrm{d}z' \,\mathrm{d}z'' \\
&= \sum_{j=1}^{S}\sum_{k=1}^{S} N_j N_k \iint [W(z,z',z'') - W_0] p_j(z') p_k(z'') \,\mathrm{d}z' \,\mathrm{d}z'' \\
&= \sum_{j=1}^{S}\sum_{k=1}^{S} N_j N_k \iint W(z,z',z'') p_j(z') p_k(z'') \,\mathrm{d}z' \,\mathrm{d}z'' - W_0 \sum_{j=1}^{S}\sum_{k=1}^{S} N_j N_k \underbrace{\iint p_j(z') p_k(z'') \,\mathrm{d}z' \,\mathrm{d}z''}_{\text{1, due to normalization (Eq. S1)}} \\
&= \underbrace{\sum_{j=1}^{S}\sum_{k=1}^{S} N_j N_k \iint W(z,z',z'') p_j(z') p_k(z'') \,\mathrm{d}z' \,\mathrm{d}z''}_{\text{original HOI term in Eq. S4}} \underbrace{- W_0 \left(\sum_{j=1}^{S} N_j\right) \left(\sum_{k=1}^{S} N_k\right)}_{\text{common factor}},
\end{aligned} \tag{S15}$$

where the last term is simply $W_0(\sum_j N_j)^2$ after relabeling summation indices. A model with a higher-order kernel where $W(z,z,z) = W_0$ is therefore equivalent to another model with $W(z,z,z) = 0$ but which has

an extra $W_0(\sum_j N_j)^2$ term. This term is z-independent, shows up for all species, and depends only on the total community density $\sum_j N_j$. Thus, whenever we have a HOI model with $W(z,z,z) = W_0$, it is understood that we subtract off W_0 from the HOI kernel and add the common density-dependent term $W_0(\sum_j N_j)^2$ to the growth rates to compensate. This maintains the principle that there is no such thing as "higher-order self-effects" even when $W(z,z,z)$ is a constant instead of zero.

Note however that this possibility no longer exists if $W(z,z,z)$ is some nonconstant function of z. In that case the extra term does not integrate out to a common factor in Eq. S15, and each species will instead pick up its own density- or frequency-dependent term. Worse, such terms can artificially promote coexistence by providing independent regulation to the species. In such cases, the real reason behind the enhanced coexistence is the presence of that extra self-regulation, and has nothing whatsoever to do with higher-order interactions. One can avoid this spurious confounding of effects by keeping $W(z,z,z)$ either zero (the most principled choice) or a constant (to be then compensated by a common density-dependent term, as detailed above). Our HOI models below all observe this constraint.

S1.3 HOIs as effective pairwise interactions

The bracketed term of Eq. S13 has the same structure as the phenotype-level growth rates of Eq. S4. Indeed, we can write $b(u)$ instead of b_i, $\alpha(u,u')$ instead of α_{ij}, and $\epsilon(u,u',u'')$ instead of ϵ_{ijk} to emphasize that they could in principle apply at any trait values, not just the u_i. Then we can write $N(u)$ instead of N_i for the same reason. This gives

$$\frac{\mathrm{d}N(u)}{\mathrm{d}t} = N(u)\left[b(u) - \int \alpha(u,u')N(u')\,\mathrm{d}u' - \iint \epsilon(u,u',u'')N(u')N(u'')\,\mathrm{d}u'\mathrm{d}u''\right] \tag{S16}$$

in place of Eq. S13. $N(u)$ may in principle either form a discrete set of finitely many Dirac deltas, or a continuous distribution. In the former case, integrating by u recovers Eq. S13. The difference is that the kernels $\alpha(u,u')$ and $\epsilon(u,u',u'')$ have now been integrated out across the intraspecific trait distributions of the participating species (Eqs. S8-S9). Eq. S16 may also be written in the form

$$\frac{\mathrm{d}N(u)}{\mathrm{d}t} = N(u)\left[b(u) - \int \alpha^{\mathrm{eff}}(u,u')N(u')\,\mathrm{d}u'\right], \tag{S17}$$

where

$$\alpha^{\mathrm{eff}}(u,u') = \alpha(u,u') + \int \epsilon(u,u',u'')N(u'')\,\mathrm{d}u'' \tag{S18}$$

is an effective pairwise interaction kernel. In discrete form, it reads $\alpha_{ij}^{\mathrm{eff}} = \alpha_{ij} + \sum_k \epsilon_{ijk}N_k$. On the surface, Eq. S17 now looks like a standard pairwise trait-based model, but with a different kernel shape that is composed of the sum of the pairwise and the higher-order terms, the latter summed over all third species taking part in the three-way interaction.

S2 Consumer-resource pairwise interaction model (evo)

In this setting, the per capita growth rates are fully determined by the phenotype z of an individual, irrespective of the species identity. These growth rates are derived from an underlying consumer-resource model (MacArthur 1970). In a standard consumer-resource model, the resource with quality y is always in quasi-equilibrium (fast dynamics compared with the population dynamics of the consumer), so its concentration, $R(y)$, is directly expressible as its maximum level,

$$R(y) = R_0(y) - \sum_{j=1}^{S} N_j \int u(z',y)p_j(z')\,\mathrm{d}z', \tag{S19}$$

where $u(z',y)$ is the degree to which a consumer individual with phenotype z' utilizes resource y and S is the total number of consumer species. The consumer per-capita growth rates then read,

$$r(z) = \int u(z,y)R(y)\,\mathrm{d}y - m(z), \tag{S20}$$

where $m(z)$ is a phenotype-specific mortality. Substituting Eq. S19 into Eq. S20, we get the standard

$$r(z) = \underbrace{\int u(z,y)R_0(y)\,\mathrm{d}y - m(z)}_{r_0(z)} - \sum_{j=1}^{S} N_j \int \underbrace{\left(\int u(z,y)u(z',y)\,\mathrm{d}y\right)}_{a(z,z')} p_j(z')\,\mathrm{d}z'. \tag{S21}$$

The above equation has the Lotka–Volterra form

$$r(z) = r_0(z) - \sum_{j=1}^{S} N_j \int a(z,z') p_j(z') \, \mathrm{d}z', \tag{S22}$$

where $r_0(z)$ is the intrinsic growth rate of trait z, and $a(z,z')$ is the interaction kernel that captures competition between trait z and z'. To determine this kernel, we first specify the resource utilization functions as Gaussian curves:

$$u(z,y) = \sqrt{\frac{2}{\omega\sqrt{\pi}}} \exp\left(-\frac{2(z-y)^2}{\omega^2}\right). \tag{S23}$$

The kernel $a(z,z')$ is then given by

$$a(z,z') = \int u(z,y) u(z',y) \, \mathrm{d}y = \exp\left(-\frac{(z-z')^2}{\omega^2}\right). \tag{S24}$$

That is, individuals with very similar traits compete strongly whereas individuals that are far apart in trait values z will compete weakly.

We want the intrinsic growth rates $r_0(z)$ in Eq. S21 to be given by a simple rectangular function along the trait axis:

$$r_0(z) = \begin{cases} 1 & \text{if } -\theta \le z \le \theta, \\ 0 & \text{otherwise,} \end{cases} \tag{S25}$$

where θ is the limit of the trait axis such that any individual which has a trait value outside of the range of $[-\theta,\, \theta]$ will have zero growth. This can be achieved with the following parameterization, among others (Pastore *et al.* 2021, Barabás *et al.* 2022): $R_0(y) = (\omega\sqrt{\pi})^{-1/2}$ is constant, and $m(z) = 1$ when $z \in [-\theta,\, \theta]$ and 0 otherwise.

Substituting Eq. S22 into Eqs. S2-S3 follows the same structure as Eqs. S13 and S14, only with the higher-order terms missing. This means that ϵ_{ijk} and γ_{ijk} are zero, and we only need to obtain the rest of the coefficients (Eqs. S7, S8, S10, and S11):

$$b_i = \frac{1}{2}\left[\mathrm{erf}\left(\frac{\theta - u_i}{\sqrt{2}\sigma_i}\right) + \mathrm{erf}\left(\frac{\theta + u_i}{\sqrt{2}\sigma_i}\right)\right], \tag{S26}$$

$$\alpha_{ij} = \frac{\omega}{\sqrt{2\sigma_i^2 + 2\sigma_j^2 + \omega^2}} \exp\left(-\frac{(u_i - u_j)^2}{2\sigma_i^2 + 2\sigma_j^2 + \omega^2}\right), \tag{S27}$$

$$g_i = \frac{\sigma_i}{\sqrt{2\pi}}\left[\exp\left(\frac{-(\theta - u_i)^2}{2\sigma_i^2}\right) - \exp\left(\frac{-(\theta + u_i)^2}{2\sigma_i^2}\right)\right], \tag{S28}$$

$$\beta_{ij} = \frac{2\sigma_i^2(u_j - u_i)}{2\sigma_i^2 + 2\sigma_j^2 + \omega^2}\alpha_{ij}. \tag{S29}$$

In Eq. S26, $\mathrm{erf}(\cdot)$ is the error function. In this model, all these quantities could be explicitly integrated out.

S3 Consumer-resource higher-order interaction model (evoHOI)

This model is the same as the previous one (evo), but with trait-mediated higher-order interactions added. The only difference is an extra term in the per capita growth rates of Eq. S22:

$$r(z) = r_0(z) - \sum_{j=1}^{S} N_j \int a(z,z') p_j(z') \, \mathrm{d}z' - \sum_{j=1}^{S}\sum_{k=1}^{S} N_j N_k \iint W(z,z',z'') p_j(z') p_k(z'') \, \mathrm{d}z' \mathrm{d}z''. \tag{S30}$$

In the last term,

$$W(z,z',z'') = \kappa \int u(z,y) u(z',y) u(z'',y) \, \mathrm{d}y \tag{S31}$$

is the higher-order interaction kernel for individuals with the three traits z, z', and z''. It is given by the three-way overlap between the resource utilization functions, and is therefore large whenever there is

substantial overlap between all three individuals' utilization curves. This means that the higher-order effects exacerbate competition whenever three individuals are adapted to consuming similar resources. The constant κ is there to make units consistent.

Eq. S31 can be integrated out:

$$W(z,z',z'') = \sqrt{\frac{4\kappa^2}{3\sqrt{\pi}\omega}} \exp\left(-\frac{(z-z')^2+(z'-z'')^2+(z''-z)^2}{3\omega^2/2}\right). \tag{S32}$$

Substituting Eq. S30 into Eqs. S2-S3 leads to equations of the form of Eqs. S13-S14. That is, apart from ϵ_{ijk} and γ_{ijk}, all integrals are as before (Eqs. S26-S29). The higher-order terms can also be integrated exactly: from Eq. S9, we have

$$\epsilon_{ijk} = \frac{2\kappa\omega^2 \exp\left(-\frac{8\left[\sigma_i^2(u_j-u_k)^2+\sigma_j^2(u_i-u_k)^2+\sigma_k^2(u_i-u_j)^2\right]+2\omega^2\left[(u_i-u_j)^2+(u_k-u_i)^2+(u_j-u_k)^2\right]}{8\omega^2\left(\sigma_i^2+\sigma_j^2+\sigma_k^2\right)+16\left(\sigma_i^2\sigma_j^2+\sigma_i^2\sigma_k^2+\sigma_j^2\sigma_k^2\right)+3\omega^4}\right)}{\sqrt[4]{\pi}\sqrt{8\omega^3\left(\sigma_i^2+\sigma_j^2+\sigma_k^2\right)+16\omega\left(\sigma_i^2\sigma_j^2+\sigma_i^2\sigma_k^2+\sigma_j^2\sigma_k^2\right)+3\omega^5}}, \tag{S33}$$

and from Eq. S12,

$$\gamma_{ijk} = -4\sigma_i^2 \frac{4\sigma_j^2(\mu_i-\mu_k)+4\sigma_k^2(\mu_i-\mu_j)+\omega^2(2\mu_i-\mu_j-\mu_k)}{8\omega^2\left(\sigma_i^2+\sigma_j^2+\sigma_k^2\right)+16\left(\sigma_i^2\sigma_j^2+\sigma_i^2\sigma_k^2+\sigma_j^2\sigma_k^2\right)+3\omega^4}\epsilon_{ijk}. \tag{S34}$$

The most straightforward interpretation of this model is that each species experiences an extra competitive effect due to three-way overlap in resource utilization, as described by Eq. S32. But following Section S1.2, one could also write Eq. S13 by defining $W'(z,z',z'') = W(z,z',z'') - 2\kappa/\sqrt{3\sqrt{\pi}\omega}$ (which is now zero when $z = z' = z''$ and negative otherwise) and adding an extra density-dependent term to compensate:

$$\frac{\mathrm{d}N_i}{\mathrm{d}t} = N_i\left[b_i - \sum_{j=1}^{S}\alpha_{ij}N_j - \sqrt{\frac{4\kappa^2}{3\sqrt{\pi}\omega}}\left(\sum_{j=1}^{S}N_j\right)^2 - \sum_{j=1}^{S}\sum_{k=1}^{S}\epsilon'_{ijk}N_jN_k\right], \tag{S35}$$

where ϵ'_{ijk} is the same as Eq. S33 but with $2\kappa/\sqrt{3\sqrt{\pi}\omega}$ subtracted off from it. Since $W'(z,z',z'')$ is nonpositive, it describes negative competition, or positive effects. Therefore, an alternative interpretation of the evoHOI model is that it is a Lotka–Volterra competition model based on resource overlap, plus an extra density-dependent term, plus a purely mutualistic three-way HOI contribution. The degree of mutualistic support depends on the dissimilarity of the phenotypes: the more dissimilar they are, the more benefit they confer to one another. The model could describe foraging herbivores who compete for food but also provide vigilance against predators, with dissimilar phenotype triplets complementing each other better (and thus conferring better protection) than similar phenotypes.

S4 The hierarchical pairwise model (hier)

Before we delve into the model details, we cite the following general integral formula (Ng & Geller 1969, integral 4.3.13):

$$\int \mathrm{erf}(z)\exp[-(az+b)^2]\,\mathrm{d}z = -\frac{\sqrt{\pi}}{a}\mathrm{erf}\left(\frac{b}{\sqrt{a^2+1}}\right), \tag{S36}$$

which, after a simple substitution of variables, reads

$$\int \mathrm{erf}(cz+d)\exp[-(az+b)^2]\,\mathrm{d}z = \frac{\sqrt{\pi}}{a}\mathrm{erf}\left(\frac{ad-bc}{\sqrt{a^2+c^2}}\right). \tag{S37}$$

We can reparameterize this as

$$\int \mathrm{erf}\left(\frac{cz-z_0}{\omega}\right)\exp\left(-\frac{(z-u)^2}{2\sigma^2}\right)\mathrm{d}z = \sqrt{2\pi}\sigma\,\mathrm{erf}\left(\frac{cu-z'}{\sqrt{\omega^2+2\sigma^2c^2}}\right). \tag{S38}$$

Using the normal trait distribution $p_i(z)$ (Eq. S1), we can rewrite Eq. S38 as

$$\int \mathrm{erf}\left(\frac{cz-z_0}{\omega}\right)p_i(z)\,\mathrm{d}z = \mathrm{erf}\left(\frac{cu_i-z_0}{\sqrt{\omega^2+2\sigma_i^2c^2}}\right). \tag{S39}$$

We now define a sigmoid interaction function as

$$a(z,\omega) = \frac{1}{2}\left[\mathrm{erf}\left(\frac{z}{\omega}\right) + 1\right]. \tag{S40}$$

This can be thought of as the convolution of the Heaviside step function with a Gaussian of width ω, which smooths out the infinitely sharp transition. Integrating this function together with the trait distribution $p_i(z)$, the result again has the form of a, but with a different point of transition and with a wider curve:

$$\begin{aligned}\int a(cz - z', \omega)p(z)\,\mathrm{d}z &= \frac{1}{2}\left[\int \mathrm{erf}\left(\frac{cz - z'}{\omega}\right)p(z)\,\mathrm{d}z + \int p(z)\,\mathrm{d}z\right] \\ &= \frac{1}{2}\left[\mathrm{erf}\left(\frac{cu_i - z'}{\sqrt{\omega^2 + 2\sigma^2 c^2}}\right) + 1\right] \\ &= a(cu - z', \sqrt{\omega^2 + 2\sigma^2 c^2}),\end{aligned} \tag{S41}$$

where we used the fact that $\int p(z)\,\mathrm{d}z = 1$ due to normalization.

Introducing the above integrals will allow us to further expand on the hierachical trait-based model. The sigmoid function of Eq. S40, written as $a(z - z', \omega)$, is a general trait-based interaction function measuring the effect of phenotype z' on z that we will use in this hierachical competition model. This now allows us to explicitly integrate all the ingredient functions of the quantitative genetic Lotka–Volterra model, given equation Eq. S22 as the per-capita growth rate of a phenotype z, with Eq. S40 as the hierachical interaction kernel. The intrinsic growth rates are chosen such that phenotypes lower in the competitive hierarchy (represented by larger values of z) have higher growth rates:

$$r_0(z) = 1 - \exp(-z/\theta). \tag{S42}$$

Given that we now have $r_0(z)$ and $a(z-z', \omega)$ at hand, and assuming $W(z, z', z'') = 0$ so that higher-order interactions are absent, we use Eqs. S7-S8 to express the ecological dynamics. When computing α_{ij} below, we apply Eq. S41 twice, with $c = 1$:

$$b_i = \int r_0(z)p_i(z)\,\mathrm{d}z = 1 - \exp\left(\frac{\sigma_i^2}{2\theta^2} - \frac{u_i}{\theta}\right), \tag{S43}$$

$$\begin{aligned}\alpha_{ij} &= \iint a(z - z', \omega)p_i(z)p_j(z')\,\mathrm{d}z'\,\mathrm{d}z = \int p_i(z)\left[\int a(z - z', \omega)p_j(z')\,\mathrm{d}z'\right]\mathrm{d}z \\ &= \int p_i(z)\,a\left(z - u_j, \sqrt{\omega^2 + 2\sigma_j^2}\right)\mathrm{d}z = a\left(u_i - u_j, \sqrt{\omega^2 + 2\sigma_i^2 + 2\sigma_j^2}\right) \\ &= \frac{1}{2}\left[\mathrm{erf}\left(\frac{u_i - u_j}{\sqrt{\omega^2 + 2\sigma_i^2 + 2\sigma_j^2}}\right) + 1\right].\end{aligned} \tag{S44}$$

These expressions are then substituted into Eq. S13 to obtain the dynamics of N_i.

Turning to the equation for the evolutionary dynamics of trait means, we first use Eq. S10 to obtain

$$g_i = \int (z - u_i)b(z)p_i(z)\,\mathrm{d}z = \frac{\sigma_i^2}{\theta}\exp\left(\frac{\sigma_i^2}{2\theta^2} - \frac{u_i}{\theta}\right), \tag{S45}$$

and then Eq. S11 to calculate β_{ij}. This has an inner integral that is the same as before in Eq. S44, and so we can write

$$\begin{aligned}\beta_{ij} &= \iint (z - u_i)a(z - z', \omega)p_i(z)p_j(z')\,\mathrm{d}z'\,\mathrm{d}z \\ &= \int (z - u_i)p_i(z)\left[\int a(z - z', \omega)p_j(z')\,\mathrm{d}z'\right]\mathrm{d}z \\ &= \int (z - u_i)p_i(z)\,a\left(z - u_j, \sqrt{\omega^2 + 2\sigma_j^2}\right)\mathrm{d}z.\end{aligned} \tag{S46}$$

To evaluate this integral, we recognize that $(z - u_i)p_i(z) = -\sigma_i^2 p_i'(z)$, where $p_i'(z)$ is the derivative of $p_i(z)$ with respect to z. So we have

$$\beta_{ij} = -\sigma_i^2 \int p_i'(z)\,a\left(z - u_j, \sqrt{\omega^2 + 2\sigma_j^2}\right)\mathrm{d}z. \tag{S47}$$

Integrating by parts, we get

$$\begin{aligned}\beta_{ij} = &- \sigma_i^2 \Big[p_i(z)\, a\Big(z - u_j, \sqrt{\omega^2 + 2\sigma_j^2}\Big)\Big]_{-\infty}^{\infty} \\ &+ \sigma_i^2 \int p_i(z)\, a'\Big(z - u_j, \sqrt{\omega^2 + 2\sigma_j^2}\Big)\, \mathrm{d}z,\end{aligned} \tag{S48}$$

where we take the derivative of a with respect to z. The first term is the product of a Gaussian and a sigmoid function, which vanishes at infinity—this term is therefore zero. In the second term, we can use the fact that the derivative of the error function is Gaussian, and the product of two Gaussians can be integrated:

$$\begin{aligned}\beta_{ij} &= \sigma_i^2 \int p_i(z)\, a'\Big(z - u_j, \sqrt{\omega^2 + 2\sigma_j^2}\Big)\, \mathrm{d}z \\ &= \frac{1}{\sqrt{\pi(\omega^2 + 2\sigma_j^2)}} \int p_i(z) \exp\left(-\frac{(z-u_j)^2}{\omega^2 + 2\sigma_j^2}\right) \mathrm{d}z \\ &= \frac{\sigma_i^2}{\sqrt{\pi(\omega^2 + 2\sigma_i^2 + 2\sigma_j^2)}} \exp\left(-\frac{(u_i - u_j)^2}{\omega^2 + 2\sigma_i^2 + 2\sigma_j^2}\right).\end{aligned} \tag{S49}$$

The dynamics of the trait means then follow Eq. S14, where g_i is given by Eq. S45 and β_{ij} by Eq. S49.

S5 The hierarchical trait-based higher-order model (hierHOI)

Including higher-order phenotypic interactions amounts to adding an extra term to the per capita growth rate:

$$\begin{aligned}r(z) = r_0(z) &- \sum_{j=1}^{S} N_j \int a(z - z', \omega) p_j(z')\, \mathrm{d}z' \\ &- \sum_{j=1}^{S}\sum_{k=1}^{S} N_j N_k \iint Wz, z', z'') p_j(z') p_k(z'')\, \mathrm{d}z'\, \mathrm{d}z'',\end{aligned} \tag{S50}$$

where $Wz, z', z'')$ is a kernel mediating three-way interactions between the phenotypes z, z', and z''. The hierarchical higher-order interaction kernel is chosen to be

$$Wz, z', z'') = \frac{\kappa}{2}\left[\mathrm{erf}\left(\frac{z_0 + z - (z' + z'')/2}{\Omega}\right) + 1\right] = \kappa a(z_0 + z - (z' + z'')/2, \Omega), \tag{S51}$$

where Ω is the width of this higher-order kernel, z_0 is an offset measuring the trait difference from z where higher-order effects start to proliferate, and κ is a constant.

All terms in Eqs. S13-S14 have already been calculated in the section above, except for ϵ_{ijk} and γ_{ijk}. The former (Eq. S9) can be obtained by repeated application of Eq. S41:

$$\begin{aligned}\epsilon_{ijk} &= \kappa a\left(z_0 + u_i - \frac{u_j + u_k}{2}, \sqrt{\Omega^2 + 2\sigma_i^2 + \sigma_j^2/2 + \sigma_k^2/2}\right) \\ &= \frac{\kappa}{2}\left[\mathrm{erf}\left(\frac{z_0 + u_i - (u_j + u_k)/2}{\sqrt{\Omega^2 + 2\sigma_i^2 + \sigma_j^2/2 + \sigma_k^2/2}}\right) + 1\right].\end{aligned} \tag{S52}$$

In turn, γ_{ijk} evaluates to

$$\begin{aligned}\gamma_{ijk} &= \kappa \iiint (z - u_i) a(z_0 + z - (z' + z'')/2, \Omega) p_i(z) p_j(z') p_k(z'')\, \mathrm{d}z''\mathrm{d}z'\mathrm{d}z \\ &= \kappa \int (z - u_i)\, a\Big(z_0 + z - (u_j + u_k)/2, \sqrt{\Omega^2 + \sigma_j^2/2 + \sigma_k^2/2}\Big) p_i(z)\, \mathrm{d}z,\end{aligned} \tag{S53}$$

after which we apply the same strategy of integration by parts that was used for β_{ij} earlier:

$$\gamma_{ijk} = \frac{\kappa\sigma_i^2}{\sqrt{\pi(\Omega^2 + 2\sigma_i^2 + \sigma_j^2/2 + \sigma_k^2/2)}} \exp\left(-\frac{(z_0 + u_i - (u_j + u_k)/2)^2}{\Omega^2 + 2\sigma_i^2 + \sigma_j^2/2 + \sigma_k^2/2}\right). \tag{S54}$$

S6 Numerical integration

We used the `deSolve` and `rootSolve` packages in R (R Core Team 2022) for numerical integration. In doing so, we proceeded in two steps. First we integrated using the backward difference method in `deSolve`'s `ode` function, for 10^{14} time units. Afterwards, to make sure about convergence, we used the final state of the time integration and used the `runsteady` function from the `rootSolve` package to integrate until all $|\mathrm{d}N_i/\mathrm{d}t|$ and $|\mathrm{d}u_i/\mathrm{d}t|$ dropped below 10^{-16}, indicating that the dynamics have converged to a fixed point. (Note: this was the case for all parameterizations in every model; we never observed eco-evolutionary cycles or chaos.)

Our model has no demographic stochasticity. This introduces the artifact that population densities can drop to arbitrarily low values and still undergo evolutionary rescue (an unrealistically tiny population evolves to the point where it can finally grow, thus surviving and establishing itself even though it was bound to go extinct). To control for this, we apply a weak Allee effect to population growth and a reduction of heritability whenever population densities are very low. We introduce

$$Q(x) = \begin{cases} 0 & \text{if } x < 0 \\ 10x^3 - 15x^4 + 6x^5 & \text{if } 0 \le x \le 1 \\ 1 & \text{if } x > 1 \end{cases} \tag{S55}$$

(Åkesson *et al.* 2021), a smooth sigmoid function which is 0 for $x < 0$, increases monotonically to one between $0 \le x \le 1$, and remains at 1 for $x > 1$ (Figure S5). Using this function, we modify the basic dynamical equations (Eqs. S2-S3) as follows:

$$\frac{\mathrm{d}N_i}{\mathrm{d}t} = Q(N_i/10^{-6})\, N_i \int r(z) p_i(z)\,\mathrm{d}z, \tag{S56}$$

$$\frac{\mathrm{d}u_i}{\mathrm{d}t} = Q(N_i/10^{-5})\, h_i^2 \int (z - u_i) r(z) p_i(z)\,\mathrm{d}z. \tag{S57}$$

That is, the right hand sides are multiplied by a factor which smoothly decreases towards zero once N_i gets below 10^{-6} and 10^{-5}, respectively.

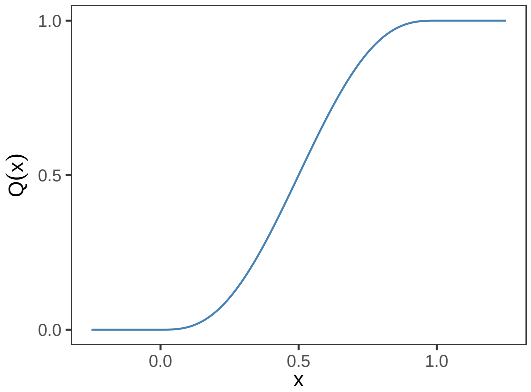

Figure S5: The smoothed step function $Q(x)$, defined by Eq. S55. It is piecewise polynomial, and twice continuously differentiable everywhere.

S7 Random structured HOIs: intraHOI

We also consider a version of the model which follows Section S2, but with ϵ_{ijk} not set to zero. Instead, they are sampled from a random uniform distribution in a way such that $\epsilon_{iij} > \epsilon_{ijk}$. In other words, intraspecific HOIs are greater than interspecific HOIs. We still assume that $\gamma_{ijk} = 0$ in this model, so the HOI terms only exert ecological but not evolutionary influence. We call this HOI model intraHOI, and evaluate the case when such a structure of random HOIs impact coexistence and trait patterning (Figures S1-S2).

S8 Community matrix, stability, and robustness of species coexistence

The Jacobian matrix J_{il} of the ecological part of the system is given by

$$J_{il} = \frac{\partial(\mathrm{d}N_i/\mathrm{d}t)}{\partial N_l}. \tag{S58}$$

The ecological dynamics follow Eq. S13. Taking the partial derivative of its right hand side by N_l:

$$\begin{aligned}\frac{\partial(\mathrm{d}N_i/\mathrm{d}t)}{\partial N_l} = J_{il} = \delta_{il}\left(b_i - \sum_{j=1}^{S}\alpha_{ij}N_j - \sum_{j=1}^{S}\sum_{k=1}^{S}\epsilon_{ijk}N_jN_k\right) \\ - N_i\sum_{j=1}^{S}\alpha_{ij}\delta_{jl} - N_i\sum_{j=1}^{S}\sum_{k=1}^{S}(\epsilon_{ijk}N_j\delta_{kl} + \epsilon_{ijk}N_k\delta_{jl}),\end{aligned} \tag{S59}$$

where δ_{ij} is the Kronecker delta ($\delta_{ij} = 1$ if $i = j$ and 0 otherwise).

The term multiplying δ_{il} in the first term is the per capita growth rate of species i (Eq. S13). When evaluating J_{il} at equilibrium, this term is zero for all persisting species. What we are left with is

$$A_{il} = -N_i\sum_{j=1}^{S}\alpha_{ij}\delta_{jl} - N_i\sum_{j=1}^{S}\sum_{k=1}^{S}(\epsilon_{ijk}N_j\delta_{kl} + \epsilon_{ijk}N_k\delta_{jl}), \tag{S60}$$

where A_{ij} is J_{ij} evaluated equilibrium—in other words, A_{ij} is the community matrix (May 1973). We can simplify this further, by performing summations with the use of the Kronecker deltas and relabeling the summation index k to j in the last term:

$$A_{il} = -N_i\alpha_{il} - N_i\sum_{j=1}^{S}(\epsilon_{ijl} + \epsilon_{ilj})N_j. \tag{S61}$$

Eq. S61 incorporates higher-order three-way interactions on top of the "usual" community matrix arising from pairwise interactions. One can then calculate the eigenvalues of this community matrix and use them to determine community stability and the robustness of species coexistence at equilibrium.

If the eigenvalues of A_{il} all have negative real parts, then the community is at a locally stable point. This means that any perturbation of the population densities at that point will decay exponentially along its eigendirection with a rate equal to the eigenvalue. The robustness of this coexistence can be calculated from the geometric mean of the eigenvalues' magnitudes (Barabás *et al.* 2016):

$$\sqrt[S]{|\lambda_1||\lambda_2|...|\lambda_S|} = \left(\prod_{i=1}^{S}|\lambda_i|\right)^{\frac{1}{S}} = \exp\left(\frac{1}{S}\log\left(\prod_{i=1}^{S}|\lambda_i|\right)\right) = \exp\left(\frac{1}{S}\sum_{i=1}^{S}\log(|\lambda_i|)\right) = \exp(\overline{\log(|\lambda|)}), \tag{S62}$$

where the overbar denotes arithmetic averaging.

S9 Ecological impacts of trait variation on species richness

Here, we present the expected ecological impact of trait variation on species richness. To this end, we make certain assumptions. One is that species heritability is zero and all species have the same trait standard deviation of σ. Previous studies have indicated that the number of species coexisting in a Lotka–Volterra model without intraspecific trait variation is proportional to inverse of the competition width, which in our pairwise Lotka–Volterra model is ω (Barabás & D'Andrea 2016, MacArthur & Levins 1967). Similarly, for a hierarchical trait-based model, the number of surviving species at the very end and the spacing between them is also proportional to the parameter ω which captures how smoothly the hierarchical competition function transitions from dominant competitors to weaker competitors in terms of their trait values.

If all intraspecific trait widths are equal, the pairwise interaction coefficients α_{ij} of the evo and evoHOI models are given by Eq. S27 after setting all $\sigma_i \equiv \sigma$:

$$\alpha_{ij} = \frac{\omega}{\sqrt{4\sigma^2 + \omega^2}} \exp\left(-\frac{(u_i - u_j)^2}{4\sigma^2 + \omega^2}\right). \tag{S63}$$

In the absence of intraspecific variation ($\sigma = 0$), this reduces further to

$$\alpha_{ij} = \exp\left(-\frac{(u_i - u_j)^2}{\omega^2}\right). \tag{S64}$$

For the hier and hierHOI models, similarly, the pairwise competition with a constant $\sigma_i \equiv \sigma$ can be written by simplifying Eq. S44:

$$\alpha_{ij} = \frac{1}{2}\left[\operatorname{erf}\left(\frac{u_i - u_j}{\sqrt{\omega^2 + 4\sigma^2)}}\right) + 1\right]. \tag{S65}$$

Without any intraspecific variation at all, this reads

$$\alpha_{ij} = \frac{1}{2}\left[\operatorname{erf}\left(\frac{u_i - u_j}{\omega}\right) + 1\right]. \tag{S66}$$

In pairwise trait-based models, the average gap between adjacent species is proportional to the width of the interaction kernel (MacArthur & Levins 1967, Szabó & Meszéna 2006, Barabás & Meszéna 2009, Barabás *et al.* 2012, D'Andrea *et al.* 2013, Barabás & D'Andrea 2016). Consequently, species richness (number of species surviving) is inversely proportional to this width. Thus, if S^* is the species richness without trait variation, then for both the eco and hier models,

$$S^* \propto \frac{1}{\omega}. \tag{S67}$$

With intraspecific trait variation that is equal across species, the new competition width is $\sqrt{4\sigma^2 + \omega^2}$ instead of ω (Eqs. S63, S65). So in the presence of trait variation, the spacing between surviving species is proportional to $\sqrt{4\sigma^2 + \omega^2}$. Thus, if S is the species richness with trait variation, then the expected predicted ratio of species richness with trait variation to species richness without can be written as

$$\frac{S}{S^*} = \frac{\omega}{\sqrt{4\sigma^2 + \omega^2}} \tag{S68}$$

Figure S6 shows this ratio as a function of trait standard deviation σ, as predicted by equation S68 and as by model simulations of the pairwise evo and hier models, with the heritabilities of all species set to zero. As seen, the theoretical curve does a good job predicting the actual ratio of species richnesses.

Comparing Eqs. S63 and S64, we see that the evo model with intraspecific variation can be usefully thought of as a model without variation, but with a wider Gaussian kernel. Doing the same comparison for the hier model leads to the same conclusion, except it is now the sigmoid function that becomes wider (Eqs. S65 and S66; Figure S3). Even more generally, we see that intraspecific variation will always have a smoothing-out effect on any interaction kernel, due to the convolution of the kernel with the Gaussian trait distributions in Eqs. S8-S9. Consequently, the main effect of increasing intraspecific trait variation is always to broaden the species-level interaction kernel and therefore limit the number of coexisting species by increasing the average space between them (Barabás & D'Andrea 2016).

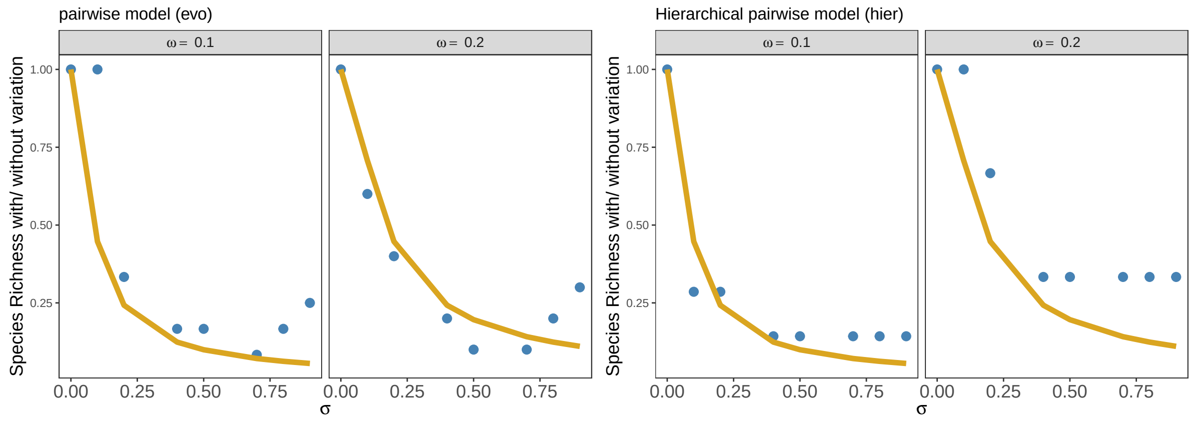

Figure S6: Ratio of species richness with trait variation to without trait variation (y-axis), plotted against trait standard deviation (x-axis) for two levels of competition widths: $\omega = 0.1$ and $\omega = 0.2$ (panels) and for two different models of pairwise competition, evo and hier (side-by-side plots). Points denote simulations of the model, while the line denotes analytical prediction based on the derivation in the supplementary section 9. The figure illustrates the negative ecological effects of trait variation on species richness.

S10 Thinking of trait-based HOIs as effective pairwise interactions

Here we explore the idea from the main text that is inspired by the form of the effective interaction kernel in Eq. S18: that a trait-based HOI model can usefully be thought of as an effective pairwise model in which the characteristic distance between adjacent species is proportional to the width of an effective interaction kernel. This kernel is in turn approximated as the sum of the kernel shapes for the pairwise and the HOI effects.

As a first step, we check whether the HOI kernel in fact behaves as one would expect; namely, that the larger its width is, the larger the spacing becomes between adjacent coexisting species and thus diversity is roughly proportional to the inverse of this width. To do this, we must isolate the HOI effects, which we do by creating a version of our models in which pairwise effects are switched off: $a(z, z') = 0$. This eliminates any purely pairwise effects from Eq. S13 by setting $\alpha_{ij} = 0$, so species only interact via higher-order terms. For additional simplicity we also set all heritabilities h_i^2 to zero—this way we only look at the ecological part of the dynamics and do not have to worry about potential confounding effects of evolution. We then reformulated Eq. S33 as

$$\epsilon_{ijk} = \frac{2\kappa\omega^2 \exp\left(-\frac{8\left[\sigma_i^2(u_j-u_k)^2+\sigma_j^2(u_i-u_k)^2+\sigma_k^2(u_i-u_j)^2\right]+2\omega^2\left[(u_i-u_j)^2+(u_k-u_i)^2+(u_j-u_k)^2\right]}{8\omega^2\left(\sigma_i^2+\sigma_j^2+\sigma_k^2\right)+16\left(\sigma_i^2\sigma_j^2+\sigma_i^2\sigma_k^2+\sigma_j^2\sigma_k^2\right)+3\omega^4+\Omega^2}\right)}{\sqrt[4]{\pi}\sqrt{8\omega^3\left(\sigma_i^2+\sigma_j^2+\sigma_k^2\right)+16\omega\left(\sigma_i^2\sigma_j^2+\sigma_i^2\sigma_k^2+\sigma_j^2\sigma_k^2\right)+3\omega^5+\Omega^2}}, \quad \text{(S69)}$$

where the only difference is the addition of Ω^2 in the denominator of the exponent and under the radical (this is what one would get by adding a constant to the utilization width). The corresponding Eq. S52 for the hierHOI model remains unchanged, because that one already depends on Ω. Now we can increase Ω from zero to larger values, which will broaden the higher-order interaction kernels. For each value of Ω, we performed 10 replicate simulations and recorded the minimum, mean, and maximum diversity. The results are in Figure S4. As seen, the models with only HOIs behave just as one would expect from pairwise models: diversity is inversely proportional to the extra width added to the kernels.

After this first step, we next switch pairwise interactions back on and check whether the model outcomes can usefully be interpreted in light of the effective, summed interaction kernels. To see wether we can do so, we make serious simplifications to remove any potential confounding factors. We set all σ_i and h_i^2 to zero, thus eliminating intraspecific variation and evolution. Furthermore, we intentionally tweak the higher-order interaction kernel from its actual form in Eq. S33 to be able to manipulate its width Ω independently of the pairwise width ω. At the species level, this results in the following interaction coefficients:

$$\alpha_{ij} = \exp\left(-\frac{(u_i-u_j)^2}{\omega^2}\right) \qquad \text{(Eq. S27 with } \sigma_i = 0\text{)}, \quad \text{(S70)}$$

$$\epsilon_{ijk} = \kappa \exp\left(-\frac{2\omega^2\left[(u_i - u_j)^2 + (u_k - u_i)^2 + (u_j - u_k)^2\right]}{\Omega^2}\right) \quad \text{(simplified Eq. S33 with } \sigma_i = 0\text{),} \quad \text{(S71)}$$

$$\alpha_{ij} = \frac{1}{2}\left[\operatorname{erf}\left(\frac{u_i - u_j}{\omega}\right) + 1\right], \quad \text{(Eq. S44 with } \sigma_i = 0\text{),} \quad \text{(S72)}$$

$$\epsilon_{ijk} = \frac{\kappa}{2}\left[\operatorname{erf}\left(\frac{z_0 + u_i - (u_j + u_k)/2}{\Omega}\right) + 1\right] \quad \text{(Eq. S52 with } \sigma_i = 0\text{),} \quad \text{(S73)}$$

This way the height and width of the pairwise kernels are 1 and ω, while those of the HOI kernels are κ and Ω, respectively. We now therefore have the ability to adjust them independently. We initialize every simulation below with 41 species of density 1 and trait u_i (which are now non-evolving and therefore constant) evenly spaced in the range $[-0.5, 0.5]$ for evo/evoHOI and in $[0, 2]$ for hier/hierHOI.

Scenario 1: $\Omega < \omega$ implies that HOIs will not contribute to diversity. The reason is that, since the pairwise kernel is broader, it already dictates the expected spacing between adjacent species. Adding a narrower HOI kernel to this will not change the effective width of their sum. Indeed, when we run the evoHOI and hierHOI models with ω twice as large as Ω, they end up almost identically to the corresponding models without HOIs (Figures S7-S8).

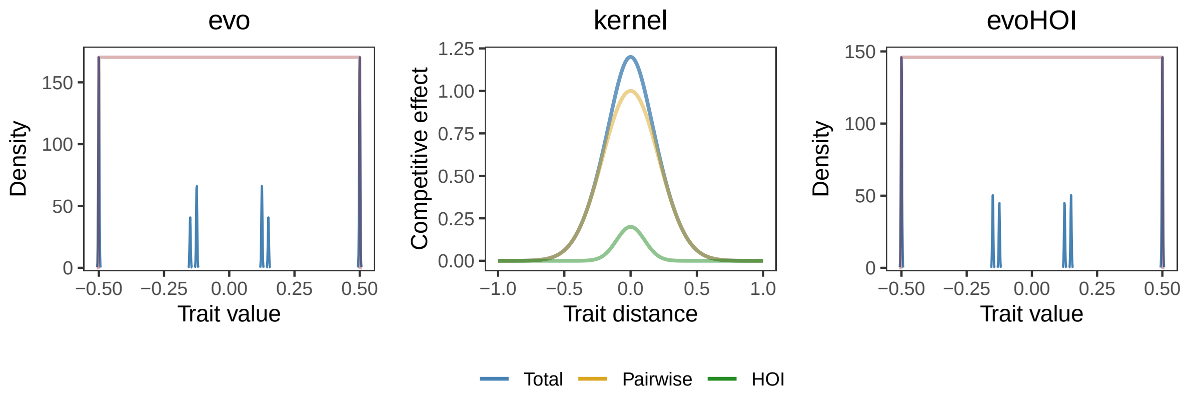

Figure S7: The middle panel, like Figure S3, depicts the shape of the pairwise part of the interaction kernel (yellow), the higher-order part (green), and their sum (blue). Since the width of the sum is visibly that of the wider pairwise kernel, we do not expect HOIs to contribute to coexistence—and they don't. The left left panel shows the final state of a community with only pairwise effects; the right panel is the final state with HOIs. Parameters: $\omega = 0.3$, $\Omega = 0.15$, $\kappa = 0.2$.

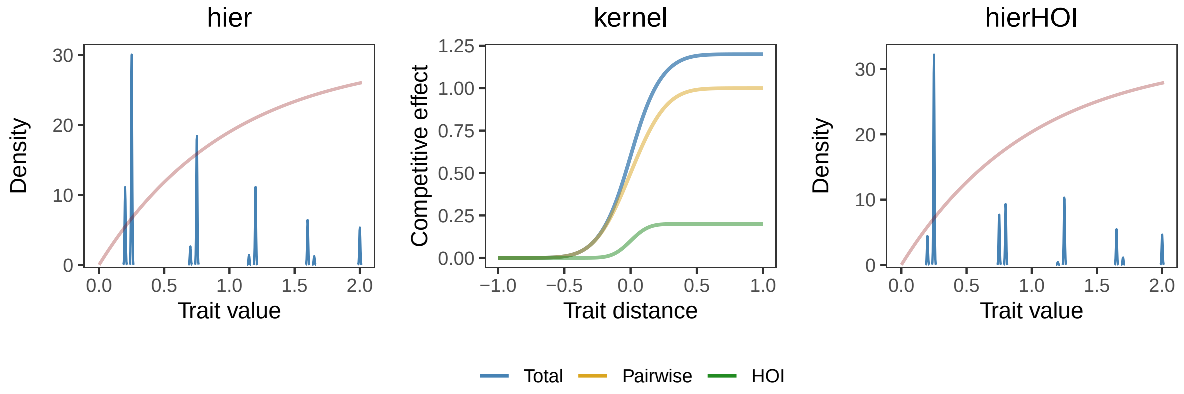

Figure S8: As Figure S7, but with the hier and hierHOI models. Parameters: $\omega = 0.3$, $\Omega = 0.15$, and $\kappa = 0.2$.

Scenario 2: $\Omega > \omega$ means that HOIs lead to diversity reduction. Now the full HOI model's kernel is broader than that of the corresponding pairwise model. Consequently, the average distance between adjacent species will also become larger in the presence of HOIs, and so we expect fewer species to survive. This is just what we observe (Figures S9-S10).

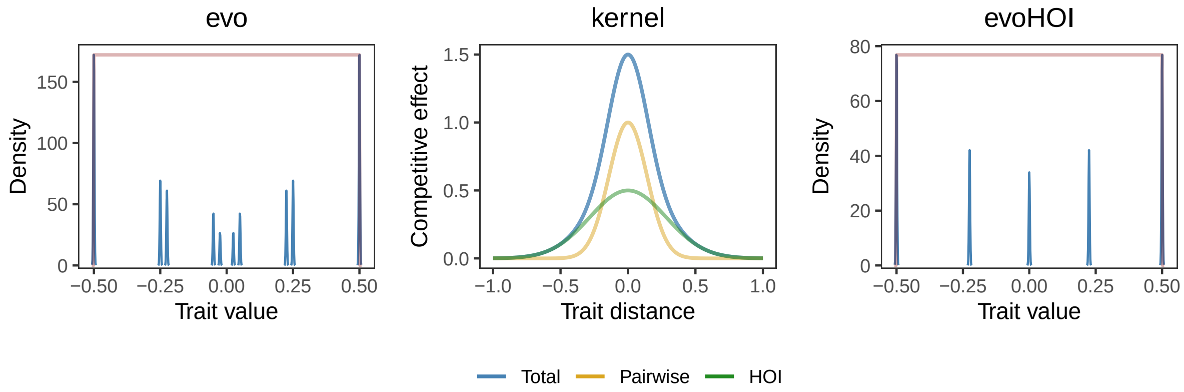

Figure S9: As Figure S7, but with $\omega = 0.2$, $\Omega = 0.4$, $\kappa = 0.5$.

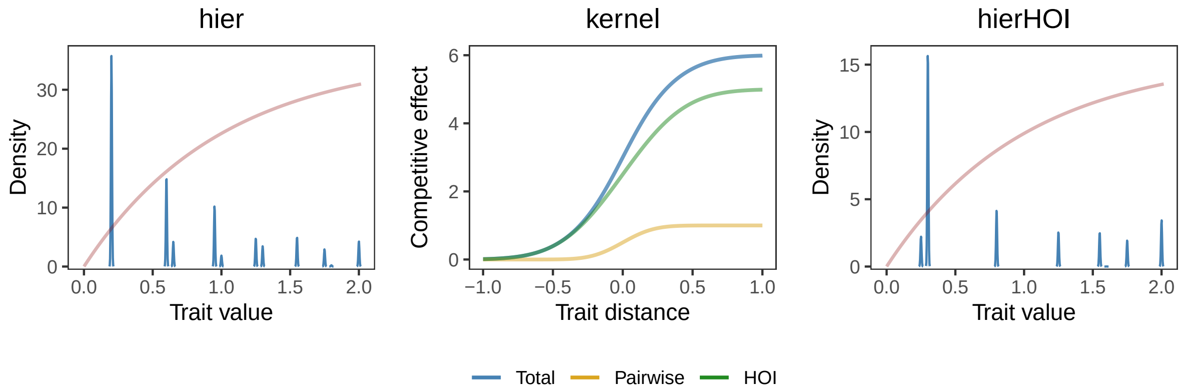

Figure S10: As Figure S7, but with the hier and hierHOI models and with $\omega = 0.25$, $\Omega = 0.5$, $\kappa = 5$.

Scenario 3: $\Omega \gg \omega$ means that HOIs no longer influence diversity. This might sound strange, since in Scenario 2 we saw that $\Omega > \omega$ leads to a reduction in diversity. But when Ω is very large, the HOI kernel becomes effectively a constant across the relevant range of the trait axis (Figures S11-S12). The width of the sum is therefore unaffected; all that happens is that the pairwise contribution is shifted vertically by a constant.

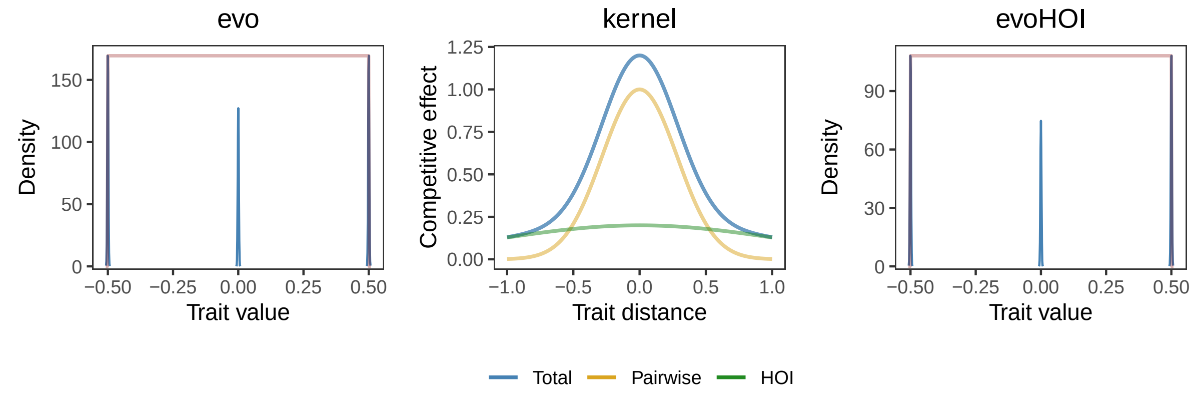

Figure S11: As Figure S7, but with $\omega = 0.4$, $\Omega = 1.5$, $\kappa = 0.2$.

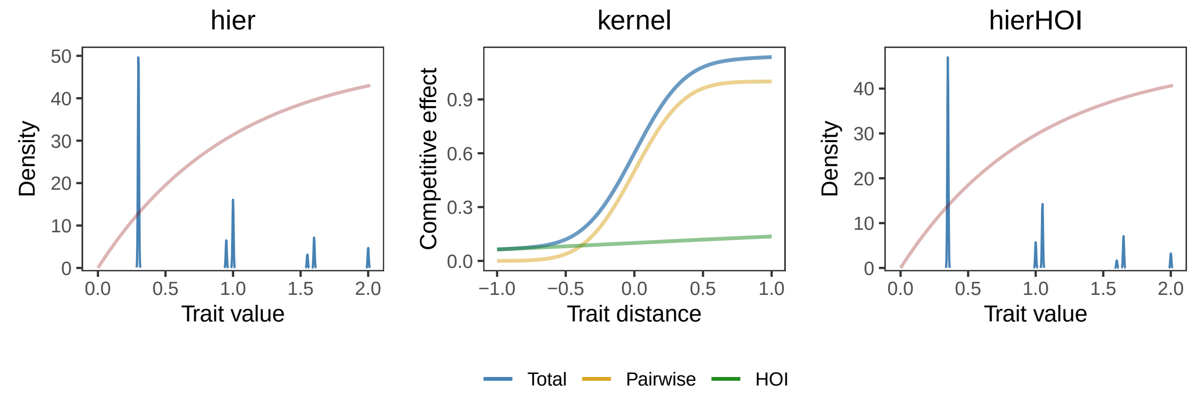

Figure S12: As Figure S7, but with the hier and hierHOI models and with $\omega = 0.4$, $\Omega = 3$, $\kappa = 0.2$.

Scenario 4: $\Omega \ll \omega$ means that HOIs increase diversity. Scenario 1 discussed the case of $\Omega < \omega$ and concluded that this does not affect diversity. How can things change then by reducing Ω even further? The answer is that when the two kernel widths are no longer comparable, we introduce two scales: a wide one and a narrow one. For the evoHOI model, since the wide kernel is approximately constant on the scale where the narrow one changes, we expect several species to coexist in clumps (Barabás *et al.* 2013, D'Andrea & Ostling 2016). This is just what we observe in Figure S13, where Ω is more than an order of magnitude smaller than ω. Conversely, for hierHOI, the sum of a wide and a very narrow sigmoid curve results in a sigmoid that itself transitions from low to high values very abruptly (Figure S14). We thus expect—and get—more diversity than without the higher-order effects.

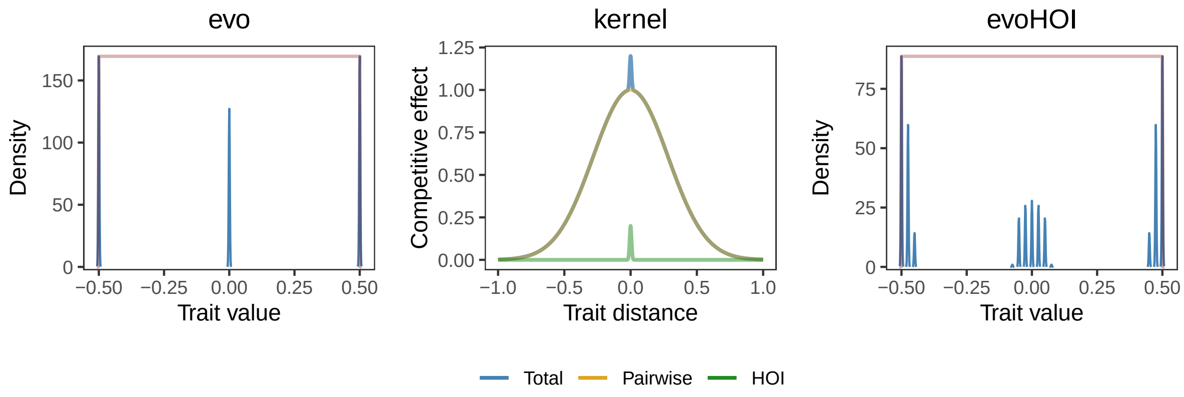

Figure S13: As Figure S7, but with $\omega = 0.4$, $\Omega = 0.01$, $\kappa = 0.2$.

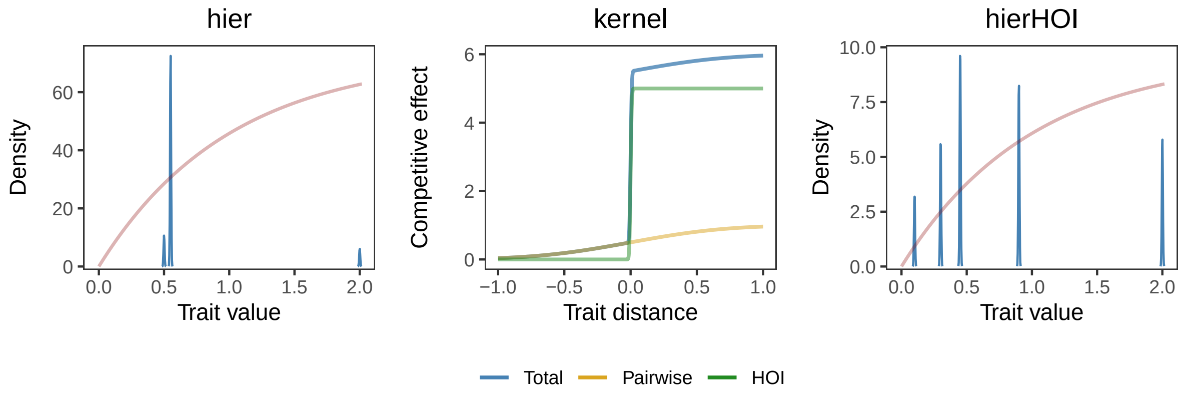

Figure S14: As Figure S7, but with the hier and hierHOI models and with $\omega = 0.8$, $\Omega = 0.01$, $\kappa = 5$.

References

Barabás, G. & D'Andrea, R. (2016) The effect of intraspecific variation and heritability on community pattern and robustness. *Ecology Letters* **19**, 977–986.

Barabás, G., D'Andrea, R., Rael, R., Meszéna, G. & Ostling, A. (2013) Emergent neutrality or hidden niches? *Oikos* **122**, 1564–1571.

Barabás, G., J Michalska-Smith, M. & Allesina, S. (2016) The Effect of Intra- and Interspecific Competition on Coexistence in Multispecies Communities. *The American naturalist* **188**, E1–E12.

Barabás, G. & Meszéna, G. (2009) When the exception becomes the rule: The disappearance of limiting similarity in the Lotka–Volterra model. *Journal of Theoretical Biology* **258**, 89–94.

Barabás, G., Parent, C., Kraemer, A., Van de Perre, F. & De Laender, F. (2022) The evolution of trait variance creates a tension between species diversity and functional diversity. *Nature Communications* **13**, 2521, number: 1 Publisher: Nature Publishing Group.

Barabás, G., Pigolotti, S., Gyllenberg, M., Dieckmann, U. & Meszéna, G. (2012) Continuous coexistence or discrete species? A new review of an old question. *Evolutionary Ecology Research* **14**, 523–554.

D'Andrea, R., Barabás, G. & Ostling, A. (2013) Revising the tolerance-fecundity trade-off; or, on the consequences of discontinuous resource use for limiting similarity, species diversity, and trait dispersion. *American Naturalist* **181**, E91–101.

D'Andrea, R. & Ostling, A. (2016) Challenges in linking trait patterns to niche differentiation. *Oikos* **125**, 1369–1385.

Lande, R. (2009) Adaptation to an extraordinary environment by evolution of phenotypic plasticity and genetic assimilation. *Journal of Evolutionary Biology* **22**, 1435–1446.

MacArthur, R. & Levins, R. (1967) The Limiting Similarity, Convergence, and Divergence of Coexisting Species. *The American Naturalist* **101**, 377–385.

MacArthur, R.H. (1970) Species packing and competitive equilibria for many species. *Theoretical Population Biology* **1**, 1–11.

May, R.M. (1973) Qualitative Stability in Model Ecosystems. *Ecology* **54**, 638–641.

Ng, E.W. & Geller, M. (1969) A table of integrals of the error functions. *Journal of Research of the National Bureau of Standards – B. Mathematical Sciences* **738**, 1–20.

Pastore, A.I., Barabás, G., Bimler, M.D., Mayfield, M.M. & Miller, T.E. (2021) The evolution of niche overlap and competitive differences. *Nature Ecology & Evolution* **5**, 330–337, number: 3 Publisher: Nature Publishing Group.

R Core Team (2022) *R: A language and environment for statistical computing.* R Foundation for Statistical Computing, Vienna, Austria.

Szabó, P. & Meszéna, G. (2006) Limiting similarity revisited. *Oikos* **112**, 612–619.

Åkesson, A., Curtsdotter, A., Eklöf, A., Ebenman, B., Norberg, J. & Barabás, G. (2021) The importance of species interactions in eco-evolutionary community dynamics under climate change. *Nature Communications* **12**, 4759, number: 1 Publisher: Nature Publishing Group.
